# Mechanistic explanation of neuroplasticity using equivalent circuits

**DOI:** 10.3389/fncom.2026.1716559

**Published:** 2026-02-13

**Authors:** Martin N. P. Nilsson

**Affiliations:** RISE Research Institutes of Sweden, Kista, Sweden

**Keywords:** adaptive filter, electric circuit, excitatory-inhibitory balance, Hebbian plasticity, homeostatic plasticity, neuroplasticity, synapse, mechanistic

## Abstract

**Introduction:**

This paper presents a comprehensive mechanistic model of a neuron with plasticity that explains how information input as time-varying signals is processed and stored. Additionally, the model addresses two long-standing, specific biological challenges: Integrating Hebbian and homeostatic plasticity, and identifying a concise synaptic learning rule.

**Method:**

A biologically accurate small-signal equivalent-circuit model is derived through a one-to-one mapping from established ion-channel properties. The often-overlooked dynamics of the synaptic cleft is essential in this process. Analysis of the model reveals a simple and succinct learning rule, indicating that the neuron functions as an internal-feedback adaptive filter, a common concept in signal processing.

**Results:**

Simulations confirm the model's functionality, stability, and convergence, demonstrating that even a single neuron without external feedback can act as a potent signal processor. The model replicates several key characteristics typical of biological neurons, which are seldom captured in other neuron models. It can encode time-varying functions, learn without risking instability, and bootstrap from a state where all synaptic weights are zero.

**Discussion:**

This paper explores the function of neurons with a focus on biological accuracy, not computational efficiency. Unlike neuromorphic models, it does not aim to design devices. The electronic circuit analogy aids understanding by leveraging decades of electronics expertise but is not intended for physical implementation. This interdisciplinary work spans a broad range of subjects within the realm of neurobiophysics, including neurobiology, electronics, and signal processing.

## Introduction

How does the brain store information? This classical question has recently received considerable attention focusing on the central nervous system's handling of coordinated synaptic changes, mandating a cell-wide coherent explanation of multisynaptic plasticity. However, a mechanistic account that links established ion-channel properties to a concise learning rule remains incomplete, despite massive experimental and theoretical efforts. This paper proposes that one route toward such an account is to map the neuron to a small-signal equivalent circuit and show that the resulting circuit implements an internal-feedback adaptive filter. The circuit is “equivalent” in the sense that it reproduces the same small-signal input–output relations for deviations around the baseline operating potential; it is not intended as a (DC) equivalent circuit for absolute voltages across compartments.

While many researchers have proposed that neurons implement adaptive filters (e.g., Widrow and Hoff, 1960; Fujita, 1982; Wolpert et al., 1998; Porrill et al., 2013; Luczak et al., 2022), to the best of the author's knowledge an explicit derivation of this function at the ion-channel level has not previously been provided.

Experimental methods typically investigate neurons' responses to stimuli and various biological manipulations such as ion channel blocking and genetic modifications. Since the first discovery of long-term potentiation (LTP) (Bliss and Lømo, 1973), experiments have revealed a diversity of overlapping and interacting plasticity mechanisms (Lisman, 2017; Sjöström, 2021). A limitation of experiments *in vitro* is that crucial parameters such as temperature, membrane potential, and calcium concentration often transcend their physiological ranges. On the other hand, experiments *in vivo* are degraded by external disturbances, such as irrelevant signals from connected neurons.

The theoretical approach is to study how plasticity *should* work. Accordingly, the principal obstacles are to identify biologically plausible mechanisms that match the theory and remain consistent with the breadth of experimental evidence. A significant theoretical contribution showed that classical Hebbian plasticity alone leads to the saturation of synaptic weights and the ensuing loss of information (Oja, 1982; Bienenstock et al., 1982). Subsequent experiments demonstrated the existence of additional, homeostatic mechanisms that prevent distortion and stabilize synaptic plasticity (Turrigiano et al., 1998; Turrigiano, 2017). Here, “homeostasis” is used in the narrow sense relevant to this model: maintaining stable *functional operation* of the neuron under changing input conditions, on the seconds–minutes time scale considered.

A useful way to relate the present work to the experimental framing of *Hebbian plasticity operating within a homeostatically maintained range* (e.g., Turrigiano et al., 1998; Turrigiano, 2017; Turrigiano and Nelson, 2004; Nelson and Turrigiano, 2008; Marder and Goaillard, 2006) is that the model does not assume distinct classes of plastic parameters implementing “Hebbian” vs. “homeostatic” components. Instead, the central claim here is that a single update rule can be intrinsically self-stabilizing: the same learning dynamics that enable associative change also enforce stability as a mathematical property of the rule itself. In the model, the only parameters that adapt are the excitatory synaptic weights, and the membrane potential is not treated as a separately tuned homeostatic variable; rather, it is a cell-wide state signal that participates directly in the weight update. Physically, this is implemented by the NMDAR pathway acting as a signed product between the presynaptic drive and the postsynaptic membrane potential, yielding a unified learning mechanism whose stability does not rely on adding a separate “range-setting” plasticity process (even though such additional processes certainly exist in real neurons).

Because of the challenges posed by the diversity of plasticity mechanisms and the scarcity of biologically plausible models, the timing and integration of homeostatic and Hebbian plasticity is an open issue (Keck et al., 2017). Therefore, a novel approach is chosen here, modeling a neuron as an electric-circuit equivalent in the spirit of Hodgkin and Huxley's seminal model (Hodgkin and Huxley, 1952) while strictly adhering to known properties of neuronal ion channels to ensure biological veracity. It should be noted that the Hodgkin-Huxley model is a model of a neuron's *axon*—specifically, the giant axon of *Loligo*. As such, it describes the *output* section of the neuron, i.e., how the neuron converts membrane potential to spike trains. In contrast, the current paper describes how the *input* section converts spike trains back to membrane potential, including an explanation of plasticity.

The classical Hodgkin–Huxley (HH) model is a deterministic, single-compartment ordinary differential equation model that accurately predicts spike waveforms. While deterministic HH dynamics can exhibit irregular (including chaotic) firing in certain regimes, the model does not explicitly represent intrinsic stochasticity (e.g., channel noise) and therefore does not, by itself, account for trial-to-trial stochastic variability of interspike intervals (ISI) under nominally identical conditions. However, the ISI probability distribution can be accurately modeled by dividing the single compartment into three distinct compartments, consisting of the distal compartment, which includes the distal dendrites; the proximal compartment, housing the proximal dendrites and the soma; and the axon initial segment (AIS) (Nilsson and Jörntell, 2021). Notably, for the analysis of the output section it is unnecessary to incorporate any considerations of synapses or plasticity. The principal mathematical difficulty of the *output* section—the proximal compartment and the AIS—lies in solving a small number of stochastic differential equations. This is undertaken in (Nilsson and Jörntell 2021), which employs the Cramér-Rao lower bound to show that the model cannot be significantly improved unless experimental data are vastly improved.

The challenges involved in analyzing the *input* section are distinctly different. A single excitatory synapse in the model is represented by around 30 coupled equations, including multiple differential equations (cf. Supplementary material). As a result, classical analysis methods become inadequate due to the excessive complexity when modeling a complete neuron. To address this, the paper leverages insights from electronics. The resulting circuit can be interpreted mechanistically as a modified *Least Mean Square* (LMS) *adaptive filter*, a versatile device well-known in the field of signal processing (Haykin, 2002; Haykin and Widrow, 2003). This interpretation takes advantage of the rich theory developed for adaptive filters. It explains precisely and quantitatively how the neuron modifies its synapses in orchestration to store time-variable functions or *signals* as required by procedural memory.

Many previous attempts to explain plasticity focus on AMPA (α-amino-3-hydroxy-5-methyl-4-isoxazolepropionic acid) receptors, assuming they not only carry the primary feed-forward signal but also control plasticity. However, this assumption leads to complications. Silent synapses, where the synaptic weight is zero, cannot convey any signal, which would cause the synapse to remain stuck at zero. Even if we assume that the synaptic weight is slightly positive, problems arise because the charge entering an AMPA receptor is directly summed into the membrane potential, making the local synapse's contribution indistinguishable from other inputs.

The above problems indicate that an additional channel appropriately reflects pre-synaptic activity, with calcium entry via NMDA (N-methyl-D-aspartate) receptors appearing to be the most viable candidate (Huganir and Nicoll, 2013; Traynelis et al., 2010; Nowak et al., 1984; Mayer et al., 1984). Whereas it is well known that variations in external calcium concentration are minuscule, the calcium flow variations being small does not pose issues in the proposed model other than slowing down adaptation because the model behaves linearly and remains stable for small excursions from equilibrium. Moreover, several experimental studies have reported that changes in extracellular calcium influence plasticity (Dunwiddie and Lynch, 1979; Turner et al., 1982; Inglebert et al., 2020; Inglebert and Debanne, 2021; Gaviño et al., 2015).

Biological experiments often require exceptionally strong stimuli to detect short-term plasticity effects. Accordingly, the experiments described here intentionally use exaggerated calcium variations to make the adaptation process more conspicuous. A more profound reason for using large variations is that significant deviations in a feedback system carry the risk of instability and functional collapse. The presented experiments demonstrate that the circuit remains stable despite substantial deflections from equilibrium.

Mechanistic models offer important advantages over empirical or purely phenomenological approaches because they aim to explain why a phenomenon occurs by specifying the interacting components and processes that generate it. Whereas empirical models primarily fit input–output relations and phenomenological models summarize observed regularities, mechanistic models delineate causal structure: they describe how one state gives rise to another through concrete interactions within the system. This emphasis on mechanism is particularly valuable for neuroscience and physiology, where explanation—not only description—is often the central goal.

Because they encode causal organization, mechanistic models also tend to generalize better to novel conditions than models that only interpolate within observed data. They provide a natural platform for hypothesis testing: one can alter a component, pathway, or parameter and predict the consequences, thereby motivating targeted experiments and reducing reliance on costly trial-and-error exploration. Mechanistic modeling further supports integration across levels of description, linking molecular and synaptic processes to cellular responses and, when appropriate, to network-level behavior. In addition, such models are useful for education and communication, because an explicit mechanism can be inspected, discussed, and shared across disciplinary boundaries. Finally, mechanistic models are well suited to iterative refinement: as new evidence accumulates, components and assumptions can be updated while preserving a coherent explanatory structure.

For these reasons, adopting a mechanistic perspective in the present work—along the lines discussed by (Craver 2006, 2007)—is important not only for accuracy and depth of understanding, but also for practical relevance. Mechanistic models bridge theory and application by providing explicit, testable accounts of how biological function could be realized.

It is tempting to include numerous ion-channel features in the model in the hope that the plasticity function will emerge automatically. In practice, however, such an approach quickly becomes unwieldy: the resulting complexity obscures which mechanisms are actually responsible for the behavior of interest and makes it difficult to determine when the model is “done.” A more productive strategy is to pursue a parsimonious construction—introducing only those channel properties that are necessary to reproduce the targeted phenomenon and deferring additional detail unless it improves explanatory power. This is particularly important because many ion channels subserve disparate roles (e.g., homeostasis, metabolism, and potentially immune-related signaling), so indiscriminately modeling them risks conflating mechanisms that are irrelevant to the plasticity function under study.

The purpose of this paper is to explain how biological neurons function, with a focus on biological accuracy. Here, computational efficiency is irrelevant. This distinction is important, as it differs from the goals of neuromorphic models, which use biological inspiration to design efficient computational devices without requiring biological accuracy. The use of an electronic circuit analogy for the neuron should not be construed as an attempt to construct a physical device. Instead, it is a method that leverages decades of experience in electronics to facilitate our understanding of the neuron's complex biophysical processes.

This paper is highly interdisciplinary, which presents challenges for readers from different academic backgrounds. Biologists are typically well-acquainted with the importance of mechanistic models but may be less familiar with signal processing principles, feedback systems, and related concepts rooted in classical engineering. In contrast, computational neuroscientists often rely on established neuron simulators and may lack exposure to foundational biological concepts such as Gray's rules or the rationale behind mechanistic modeling.

Additionally, modeling in terms of active electronic components is uncommon in both fields, yet this perspective is essential for the present work. The biological, signal processing, and electronic aspects are all critical and cannot be excluded without compromising the integrity of the overall approach.

To support a broad readership, care has been taken to include sufficient detail across these domains. Naturally, some readers may find certain sections overly detailed, while others may consider the same material insufficient. Given space constraints, a balance has been sought. For those requiring additional background, the following baseline resources are recommended: Purves et al. (2012), Ch. 1–8) for neurobiology, (Horowitz and Hill 1989, Ch. 1–7) for electronics, and (Widrow and Stearns 1985, Ch. 1–12) for adaptive signal processing.

### Organization of this paper

The paper's main topic is a derivation of the equivalent circuit and adaptive filter model from established knowledge about neuronal ion channels. For a mechanistic model, it is imperative to select an appropriate level of description that is adequately detailed yet not overly complex to provide a functional explanation and address the three specific problems under consideration. To achieve this, the paper first reviews the established function of inhibitory and excitatory synaptic ion channels to a level that allows for a direct translation into an electric network. By this conversion, insights from a century of experience with electronic circuits can be leveraged, along with the ability to identify circuit patterns or “motifs.” The approach is conservative in not assuming the existence of as-yet-undiscovered biological mechanisms.

The paper's main conclusion is that a single neuron can be abstractly and mechanistically characterized as an adaptive filter, a powerful and fundamental component in signal processing. The basic principles of adaptive filters are, therefore, briefly reviewed. An adaptive filter's function in its fullest generality is to determine how a reference input is expressible in terms of a given set of input components.

Four experiments are performed to further support the claim that the neuron operates as an adaptive filter. The first experiment demonstrates the circuit's ability to adjust excitatory synaptic weights so that the weighted excitatory drive approximates (balances) the inhibitory reference signal. It also confirms that presynaptic action potentials act as clock pulses (“strobes”) that trigger synaptic weight updates. The second experiment examines the model's behavior under redundant (linearly dependent) inputs and tests whether the synapse model can be interpreted as a lumped aggregate of biological presynaptic neurons without introducing instability. The third experiment initializes the excitatory weights to overly large values and shows that the same learning rule automatically reduces (depresses) the weights, converging to the same solution obtained when the weights are initialized at zero. The fourth experiment visualizes the time course of the prediction-error feedback, making the bidirectional evolution of the prediction error during learning directly observable.

The Results section presents the convergence and stability outcomes of the experiments diagrammatically, followed by an explanation of how the model, in its adaptive filter capacity, addresses the three specific issues: information storage and retrieval, Hebbian-homeostatic plasticity, and the synaptic learning rule.

Subsequently, the discussion section introduces related work and explores some implications of viewing the neuron as an adaptive filter.

The investigation spans a time frame from milliseconds to minutes, encompassing short-term plasticity (STP) and early long-term potentiation (LTP) while excluding late LTP due to its reliance on nuclear processes and its consolidating function.

The neuron model introduced here lays the groundwork for a more complex mechanistic model that examines neuron populations and their coding mechanisms. However, creating a model encompassing large networks of neurons requires sophisticated signal processing techniques, such as wavelet decomposition and the concept of sparsity. These aspects are beyond the scope of the current paper but are detailed in a separate study (Nilsson, 2023). To summarize that study briefly, it demonstrates how populations of neurons conforming to the adaptive-filter model discussed here can effectively transmit, process, and store information. This is achieved through an invariance property, which can be geometrically characterized as a convex cone. The adaptive filtering characteristics of these neurons enable them to perform signal processing tasks compactly and efficiently. An algebra of convex cones can abstractly describe these operations. This provides the populations with a robust computational framework akin to a “programming language” for neurons.

In summary, this article models a neuron's primary biochemical information processing pathways as equivalent electric circuits, reviews the adaptive filter concept, and employs it to describe the neuron's overall function. The model's adequacy is demonstrated through four simulation experiments, substantiating the neuron's capacity to operate as an adaptive filter. These results support the proposed model's validity and potential for advancing research in this field, demonstrated by its application as a foundation for a mechanistic model of neuron populations (Nilsson, 2023).

## Modeling the neuron

### Overall structure of a neuron

This subsection provides a detailed description of the structure and function of a neuron, highlighting its key components, synaptic types, and their roles in signal transmission and plasticity.

The target neuron is a generic, glutamatergic neuron equipped with AMPA receptors (AMPARs) and NMDA receptors (NMDARs). This kind of neuron has been extensively studied and is representative of a substantial fraction of neurons in the central nervous system (CNS) (Traynelis et al., 2010), typical examples of which are the hippocampal neurons where LTP was first demonstrated (Bliss and Lømo, 1973).

**Figure 1:**
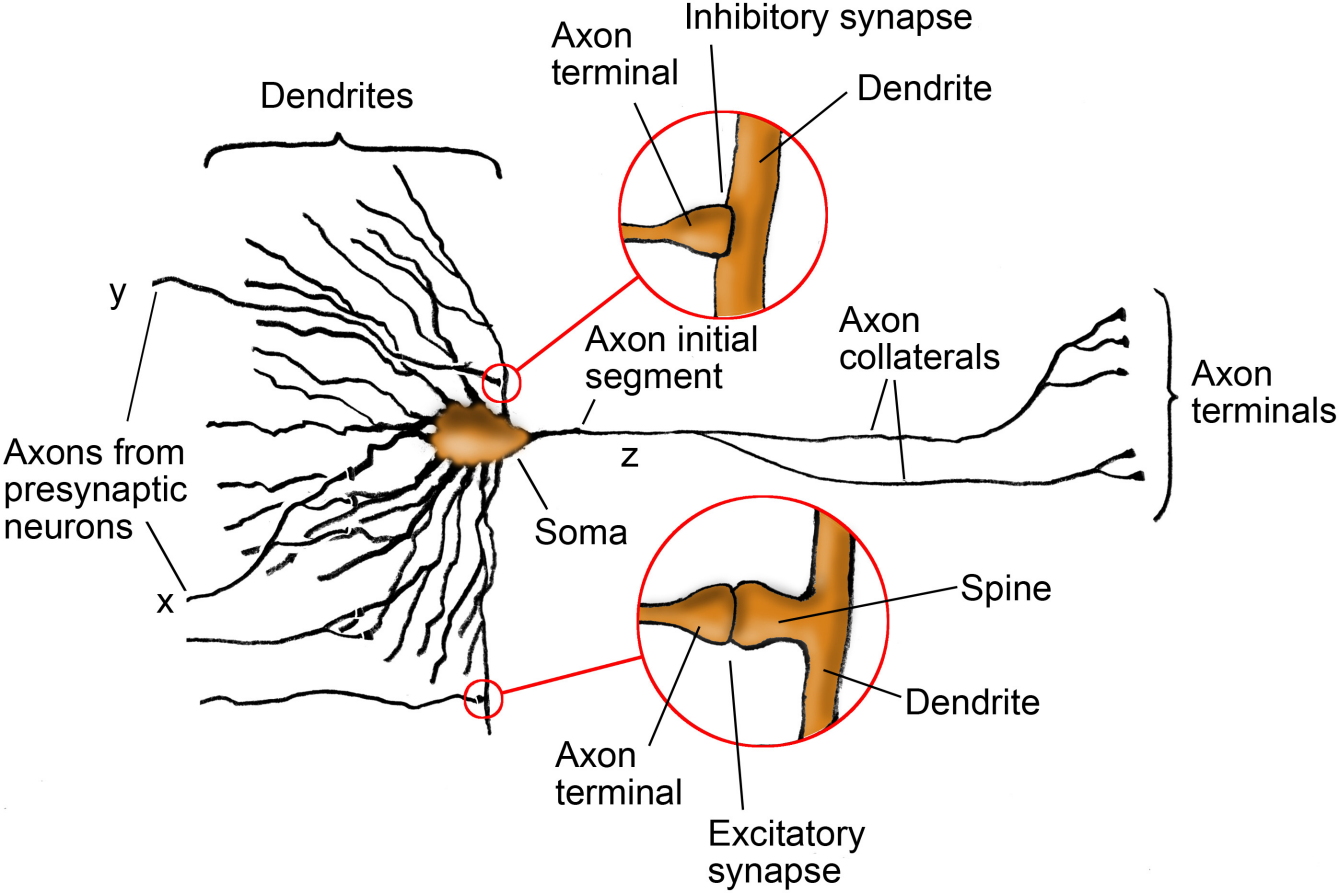
Structure of a generic neuron. This image overviews the essential parts of a neuron, with details of inhibitory and excitatory synapses explored in figures below.

The primary components of a neuron include the dendrites, which receive inputs from presynaptic neurons; the soma, which aggregates the contributions from dendrites; and the axon, which transmits the result to other neurons (Figure 1). Axons can branch into axon collaterals carrying identical signals. Synapses, the contact points between axons and dendrites, are of two types: inhibitory and excitatory. They convert incoming stochastically rate-coded sequences of action potentials (APs), or more tersely, PFM (pulse-frequency modulated) spiketrains (Nilsson and Jörntell, 2021), into postsynaptic currents that alter the membrane potential, the voltage difference between the neuron's interior and exterior. At the axon initial segment (AIS), this potential is converted back into a spiketrain for output via the axon.

Ultrastructural studies show that inhibitory synapses are typically located proximally, directly on dendritic shafts or the soma, whereas excitatory synapses are more often situated distally on dendritic spines. Because spines are strongly associated with structural and functional plasticity, this anatomical asymmetry has historically motivated the heuristic that excitatory synapses are the primary locus of synaptic plasticity, while inhibitory synapses are treated as comparatively fixed—an idea sometimes referred to as Gray's rules (Gray, 1959; O'Brien et al., 1998; Traynelis et al., 2010; Harris and Weinberg, 2012).

This work adopts Gray's rules only as a modeling prior, i.e., as a pragmatic asymmetry that guides where we place the model's explicit learning mechanism, not as a biological claim that inhibitory synapses are intrinsically non-plastic. While it is well established that GABAA synapses can express plasticity, the corresponding induction pathways are diverse and strongly context-dependent, and—crucially for our purposes—there is no single, canonical “NMDA-like” coincidence gate at GABAA synapses that would naturally yield a unique, voltage-dependent learning rule analogous to NMDAR magnesium unblock–controlled calcium influx. Given that the model focuses on a seconds–minutes time window, we therefore treat GABAA synaptic efficacies as effectively constant during the modeled episodes, interpreting them as pre-tuned parameters (potentially reflecting slower regulatory processes outside the scope of the model). Most importantly, we deliberately avoid introducing an arbitrary inhibitory plasticity rule: without a principled, uniquely motivated induction mechanism for the inhibitory synapses in this setting, adding such a rule would primarily increase the model's degrees of freedom and risk overfitting, without improving the explanatory power for the plasticity phenomenon we aim to capture.

### Inhibitory synapse mapping

This subsection describes how a biological synapse responds to a presynaptic action potential, including the roles of neurotransmitter release and postsynaptic receptor activation. It then presents the corresponding equivalent electric-circuit representation of this process, in the spirit of Hodgkin and Huxley's axon model (Hodgkin and Huxley, 1952).

**Figure 2:**
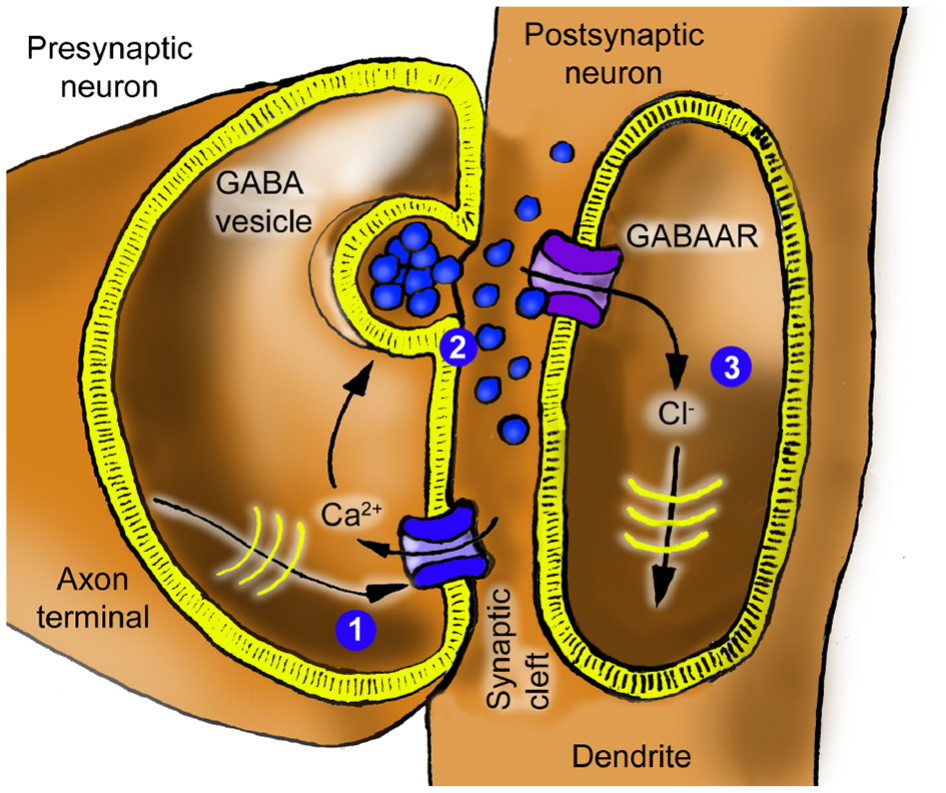
Inhibitory synapse schematic. An action potential arriving at (1) causes the release of GABA at (2), which then activates GABAAR, allowing a chloride current at (3), traveling as an IPSC to the soma.

When action potentials reach the axon terminal (Figure 2), the membrane potential depolarizes (increases), causing voltage-gated calcium channels (Ca_*V*_) to open (1). The calcium ion influx triggers the release of the neurotransmitter γ-aminobutyric acid (GABA) from nearby vesicles (2) into the synaptic cleft. GABA binds to GABA type A receptors (GABAAR) on the postsynaptic neuron, opening the receptor channel to chloride ions (3) (Sallard et al., 2021). These ions are negatively charged and hyperpolarize (reduce) the membrane potential.

The biological synapse is mapped to an electric circuit as follows: both neurotransmitter and ion flow are modeled as electrical currents. In addition to representing electrical potential, voltages are used to represent accumulated quantities and concentrations (e.g., ions) through the circuit analogy.

The model is formulated as an AC (small-signal) analysis: all voltages represent deviations from a quiescent operating point (baseline potential) *E*_quiesc_. Specifically, for any membrane or compartment potential *V*(*t*) we work with the deviation variable *U*(*t*) = *V*(*t*)−*E*_quiesc_. The ground symbols in the schematics denote the single global reference *U* = 0, i.e., the quiescent baseline, and are not meant to indicate a physical ground located in any specific anatomical region. Using one common reference across compartments is a deliberate simplification enabled by the AC restriction: DC offsets between compartments are outside the scope of the model and are not represented.

In the model, presynaptic transmitter release is represented as a pulse waveform applied to the “gate” input of ideal behavioral channel elements (Equation 1). This gate waveform should be interpreted as a normalized gating proxy (e.g., transmitter concentration/open-probability drive), expressed in volts only as a simulation convenience; it is not a physical bias voltage comparable to ion reversal potentials. Its absolute amplitude therefore constitutes a scaling convention: multiplying the gate waveform by a factor *a* can be compensated by dividing the corresponding Gain parameter γ by *a* (or by *a*^2^ when two gates are multiplied), leaving the resulting currents and dynamics unchanged. The 100 mV pulse amplitude used in the experiments was chosen for numerical convenience, and the model does not rely on it, nor does it imply saturation of channel conductance in the semiconductor sense.

**Figure 3:**
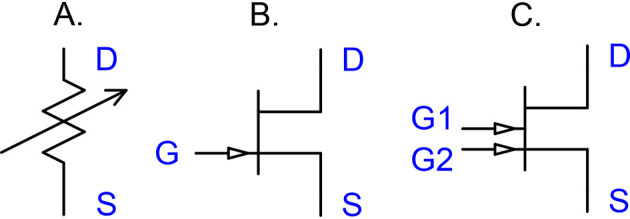
Gated ion channels are transistors. Gated ion channels are effectively FET transistors. **(A)** Hodgkin and Huxley used a variable resistance to describe gated ion channels. In their model, the gating connection was not drawn explicitly but appeared in the constitutive equation for the channel. **(B)** Today, the transistor concept is well-established and, in particular, an ideal FET (field effect transistor) is an excellent model for a gated ion channel. A voltage-gated calcium channel Ca_*V*_ is accurately described by a transistor where the gate voltage *V*_*G*_ is provided by the membrane potential, controlling the drain-source (DS) current *I*_*DS*_ by the constitutive equation *I*_*DS*_ = γ*V*_*G*_*V*_*DS*_. Similarly, a transistor can be used for the GABAAR, where the gate voltage models the GABA concentration. **(C)** Double-gated transistors appropriately model NMDAR and AMPAR defined by *I*_*DS*_ = γ*V*_*G*1_*V*_*G*2_*V*_*DS*_, involving the two gating inputs *G*1 and *G*2. For the NMDAR, these are the glutamate concentration and the postsynaptic membrane potential, respectively, whereas for the AMPAR, they are the glutamate concentration and the number of AMPAR receptors, respectively.

The model adopts Hodgkin and Huxley's view of gated ion channels as voltage-controlled conductances (Figure 3A). Because of their similarity to *ideal* field-effect transistors (FETs), the schematic uses modern transistor symbols (Figures 3B, C). A formal rationale that a local population of gated ion channels can be identified as a transistor is that they are governed by the same constitutive equation,


$$\begin{array}{l} I_{\text{channel}}=\gamma V_{\text{gate}}V_{\text{channel}}, \end{array}$$ (1)

saying that the channel current is proportional to the product of the gate and channel voltages, where the factor γ*V*_gate_ denotes the total channel conductance.

**Figure 4:**
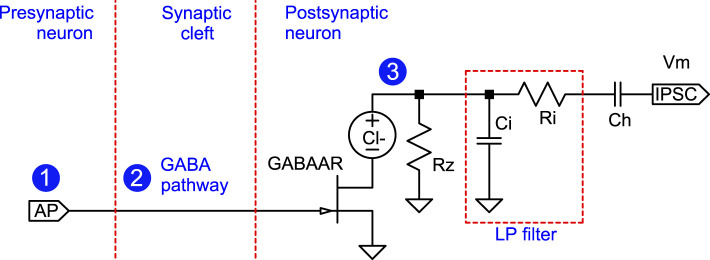
Inhibitory synapse circuit equivalent. This circuit implements the inhibitory synapse described in Figure 2 by modeling neurotransmitters as electrical currents. The transistor represents GABA-gated ion channels. Locations indicated by circled numbers 1–3 correspond to identically marked locations in Figure 2. As long as the membrane potential is higher than the reversal potential of chloride, which is normally the case, a positive pulse input at AP will lead to a negative current pulse at IPSC. The capacitor *C*_*h*_ models the cellular machinery retaining homeostatic equilibrium. The transistor symbol denotes an *ideal* FET-like bidirectional voltage-controlled conductance defined by Equation 1 (not a physical n-channel MOSFET). Current direction is determined by the sign of the driving force across the channel. The polarity markers on the voltage sources indicate the sign convention/reference direction for the reversal potentials used in Table 1 and the equations, not a fixed physical current direction. The ground symbol denotes global AC reference; not a physical ground.

The GABAAR is defined by the equation *I*_*DS*_ = γ*V*_*G*_*V*_*DS*_, where the constant γ = γ_GABAAR_ is the transistor's gain, and *I*_*DS*_ and *V*_*DS*_ = *V*_*D*_−*V*_*S*_ are the channel current and voltage, respectively, between the transistor's *D* (“drain”) and *S* (“source”) terminals or “pins.” *V*_*G*_ is the gate voltage representing the GABA concentration. The voltage source “Cl-” represents the offset of the quiescent potential *E*_quiesc_ from the reversal potential *E*_Cl_ for chloride ions, so


$$\begin{array}{l} I_{\text{GABAAR}}=I_{DS}=\gamma V_{G}V_{DS}\\ \;=\left(\gamma V_{G}\right)\left(V_{D}-V_{S}\right)=g\left(V_{G}\right)\left(V_{m}-E_{\text{Cl}}\right), \end{array}$$ (2)

recognizable as the traditional equation for the ion channel current where *g*(*V*_*G*_) is the conductance, *V*_*m*_ is the local membrane potential at (3) in Figure 4, and *V*_*m*_−*E*_Cl_ is the electrochemical driving force.

Reversal potentials *E*_ion_ are distinct from the quiescent operating point *E*_quiesc_. Ion-specific reversal potentials should not be conflated with the quiescent *E*_quiesc_, which is solely the reference for the AC deviations. In other words, *E*_quiesc_ defines the origin for the (local) membrane potential deviation, whereas *E*_ion_ defines the direction and magnitude of ionic currents.

A single transistor is chosen to represent the entire population of GABAARs at one synapse. Overall, the circuit inverts an incoming train of positive voltage pulses to negative current pulses and filters them through a lowpass filter before integrating them into the membrane potential.

The resistor *R*_*z*_ represents the transport processes that circulate the chloride back out of the cell. The signal is filtered on its way to the soma by a lowpass filter *R*_*i*_*C*_*i*_ composed of the spino-dendritic axial resistance *R*_*i*_ and capacitance *C*_*i*_. The filter properties of synapses can vary depending on their proximity to the soma. In the current inhibitory synapse model, this variability can be represented by adjusting the *R*_*i*_ and *C*_*i*_ components. Nonetheless, to maintain simplicity in the explanation, this feature is not used in the example scenarios below.

In the present AC formulation, synaptic “delays” are modeled as causal kinetics (phase lag arising from low-/high-pass dynamics) rather than as pure transport delays. The RC elements therefore explicitly encode timing through their time constants and associated frequency-dependent phase shifts. Fixed conduction delays are neglected because they add an unconstrained constant phase factor and do not change the qualitative learning mechanism on the modeled timescale.

Because the relevant internal variables are generated by first-order lowpass filtering with time constant τ, the circuit is largely insensitive to the fine temporal details of presynaptic waveforms on sub-τ timescales. In this regime, the lowpass output is primarily determined by the recent time integral of the input over a window of order τ. Because the relevant internal variables are generated by first-order lowpass filtering with time constant τ, the circuit is largely insensitive to the fine temporal details of presynaptic waveforms on sub-τ. Consequently, a longer-duration presynaptic signal is well approximated by a sequence of short, stereotyped pulses: what matters most is the total pulse area accumulated within roughly one time constant, rather than the precise pulse shape. In the simulations we therefore represent presynaptic events as triangular pulses of 1 ms duration, and longer effective waveforms as trains of such pulses; this changes the cumulative drive to the filtered traces (an input-dependent gain effect) without constituting a separate long-term plasticity mechanism.

The series capacitance *C*_*h*_ effectively encapsulates the homeostatic mechanisms that stabilize the neuron's internal potential at a biologically optimal level. Alternatively, it can be viewed as a means of compartmentalizing the neuron's functional regions. In a single-compartment neuron model, the membrane resistance *R*_*m*_ and membrane capacitance *C*_*m*_ largely determine the neuron's frequency characteristics. However, as demonstrated in (Nilsson and Jörntell 2021), a mechanistic explanation of spike generation requires at least three distinct compartments. In this framework, the resistor *R*_*z*_ serves to establish a local reference potential for the distal compartment and the *R*_*i*_ and *C*_*i*_ its impedance. The output of the inhibitory synapse into the dendrite or soma is a negative current pulse, the inhibitory postsynaptic current (IPSC).

### Excitatory synapse mapping

This subsection examines the functioning of an excitatory synapse, including the roles of calcium ions, glutamate, AMPARs, NMDARs, synaptic plasticity, and the translation of these biological processes into an equivalent electric-circuit model.

**Figure 5:**
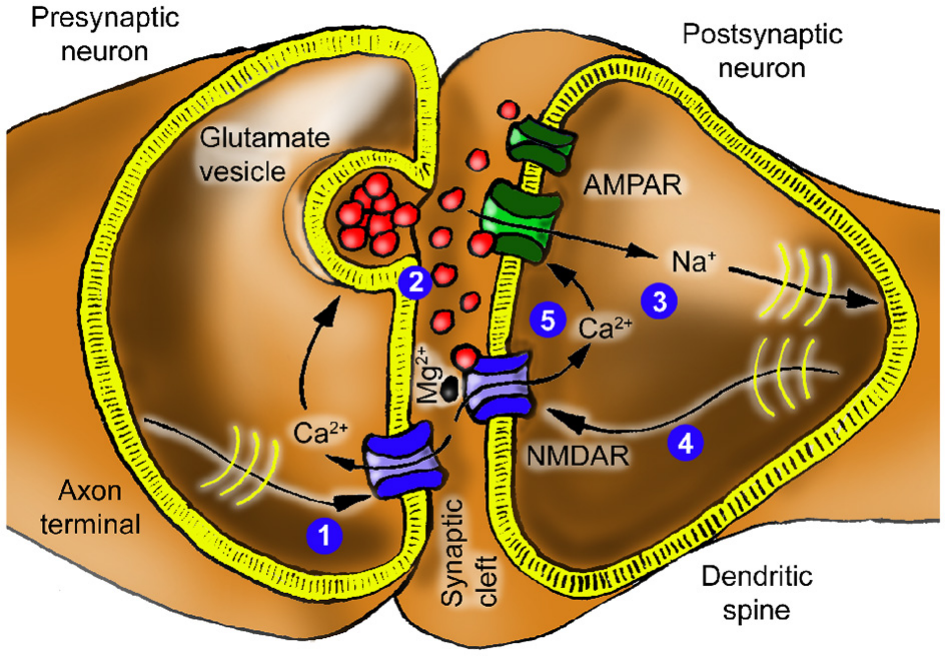
Excitatory synapse schematic. An action potential arriving at (1) causes the release of glutamate at (2), which then activates AMPARs, allowing a cation current at (3), here represented by sodium ions. This current travels to the proximal dendrites and soma, where it is lowpass filtered and fed back as the membrane voltage (4). At (5), this voltage and glutamate gate the NMDAR. Mg^2+^denotes the extracellular magnesium block of NMDARs, which varies with the membrane potential *V*_*m*_. Calcium influx is shown both presynaptically (via voltage-gated Ca^2+^ channels, triggering release) and postsynaptically (via NMDAR when Mg^2+^ block is relieved). Experiments have demonstrated the activity-dependence of the synaptic cleft's calcium concentration ${\left[{\text{Ca}}^{2+}\right]}_{e}$ (Borst and Sakmann, 1999; Cohen and Fields, 2004).

The function of an excitatory synapse (Figure 5) is similar to that of an inhibitory synapse, but the plasticity associated with spines adds complexity to the model. After lowpass filtering, excitatory input pulses increase the postsynaptic membrane potential, and the synapse's gain is modified depending on the input's magnitude and the current membrane potential.

In more detail, the arriving action potential enables calcium ions to enter the presynaptic terminal (1) and trigger the release of the neurotransmitter glutamate (2). Glutamate binds to AMPARs on the postsynaptic neuron, opening the channels to positively charged sodium ion inflow and potassium ion outflow (3); the resulting net inward current depolarizes the membrane potential. The model opts for simplicity by depicting sodium ions only. Glutamate also affects NMDA receptors involved in the neuron's plasticity. The NMDA receptor is distinguished by its gating mechanism (Huganir and Nicoll, 2013; Traynelis et al., 2010). While the binding of glutamate is essential, it alone is insufficient to open the channel. A magnesium ion is a gatekeeper that blocks the channel in a graded relation to the neuron's membrane potential (Nowak et al., 1984; Mayer et al., 1984). Depolarization of the neuron removes this magnesium block (4), enabling calcium to flow through the NMDA receptor channel (5). This calcium influx regulates *the number* of AMPA receptors constituting the synaptic weight through a cascade of downstream reactions (O'Brien et al., 1998; Huganir and Nicoll, 2013).

**Figure 6:**
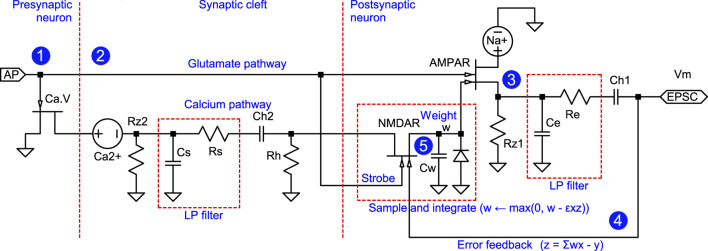
Excitatory-synapse circuit equivalent. In analogy with Figure 4, the above circuit models the excitatory synapse described in Figure 5 by representing the flow of neurotransmitters as electrical currents. Transistors represent gated ion channels. Locations indicated by circled numbers 1–5 correspond to identically marked locations in Figure 5. An action potential arriving at (1) causes the release of glutamate at (2), which then activates AMPARs, allowing a sodium current at (3). This current travels to the proximal compartment, where it is lowpass filtered and fed back as the membrane voltage (4). At (5), this voltage and glutamate gate the NMDAR transistor. Together with the capacitor *C*_*w*_, it represents the downstream cascade, eventually integrating the signal into the number of AMPARs. The annotations clarify the function of the components and indicate the adaptive filter function in analogy with Figure 7. The calcium pathway and the sample-and-integrate block are essential to the model, as NMDAR-mediated calcium influx regulates the number of AMPARs and thereby defines the synaptic weight. The capacitors *C*_*h*1_ and *C*_*h*2_ and the resistor *R*_*h*_ represent the cellular machinery retaining homeostatic equilibrium. The function of the NMDAR is based on the deviations from this equilibrium (“AC-analysis”). The ground symbol denotes global AC reference; not a physical ground. The signals *x* (excitatory drive) and *y* (inhibitory drive) are defined at the more abstract level shown in Figure 7; they are implemented as spiketrain PFM modulations and are not intended as labels of specific circuit-node voltages in this schematic.

The source of calcium ions is the synaptic cleft. This calcium is consumed *both* by the presynaptic terminal via the Ca.V channel *and* the NMDA channels via the “calcium path” illustrated in Figure 6, which shows an electric-circuit equivalent for the excitatory synapse. Again, this is a direct translation of the biochemical processes of the excitatory synapse in Figure 5. Similar to the “Cl-” voltage source in the inhibitory synapse circuit, the “Ca2+” and “Na+” voltage sources represent the differences between the quiescent potential and the reversal potentials for calcium and sodium, respectively.

Figure 6 is a small-signal (AC) equivalent circuit: sources corresponding to constant electrochemical gradients are absorbed into the operating point and are not shown explicitly. Capacitors *C*_*h*1_ and *C*_*h*2_ provide DC isolation between compartments, preventing baseline (DC) gradients/steady currents from entering the AC network. This is a modeling abstraction for analyzing deviations that drive plasticity signals and should not be interpreted as a literal DC physiological depiction of where ionic gradients are stored.

A voltage pulse (1), representing the presynaptic action potential and corresponding glutamate release (2), gates injection of a positive current through the AMPAR. This current (3) is lowpass filtered as it travels to the soma, with a cutoff frequency of *f*_*c*_ = 1/(2π*R*_*e*_*C*_*e*_), which can vary significantly between different synapses within the same neuron. The filtering characteristics of excitatory synapses, much like those of inhibitory synapses, are influenced by their spatial positions and can be modeled using the *R*_*e*_ and *C*_*e*_ components. The culmination of this process is an excitatory postsynaptic current (EPSC) that is integrated with other EPSCs and inhibitory postsynaptic currents (IPSCs) by the membrane capacitance *C*_*m*_ of the soma and proximal dendrites. The ensuing (AC, alternating current) membrane potential *V*_*m*_ is electrotonically propagated throughout the cell (4).

The calcium concentration in the synaptic cleft is depleted when a presynaptic action pulse arrives, because the pulse causes calcium channels to open, consuming some of the synaptic cleft's calcium content (Borst and Sakmann, 1999; Cohen and Fields, 2004). This reduction of ${\left[{\text{Ca}}^{2+}\right]}_{e}$ upon activity in the synaptic cleft reduces the driving force for calcium entry through the NMDAR, which plays a crucial role in the mechanism underlying synaptic enhancement (Bliss and Collingridge, 1993).

The cluster of NMDARs, modeled here as a dual-gate transistor, senses the membrane potential with one gate, whereas the other (“strobe”) recognizes glutamate activation, enabling synaptic weight modification. The synaptic cleft acts as a calcium buffer and effectively lowpass filters the calcium-encoded signal with a time constant given by *R*_*s*_*C*_*s*_. In this context, “buffer” refers to a reservoir with large—but finite—capacity. As a result, perturbations such as tapping into it still produce noticeable effects.

The voltage across the capacitor *C*_*w*_ represents the synaptic weight, which governs the variable number of AMPARs. In this model, the cluster of AMPARs is represented by a dual-gate transistor, enabling modulation by NMDARs. Since the number of AMPARs must be non-negative, the voltage across *C*_*w*_ is constrained to be non-negative by a diode connected in parallel with *C*_*w*_.

The NMDARs' key role is to act as a *multiplier* of deviations *x* and *z* from baseline in presynaptic activity and error feedback, respectively. The external calcium concentration ${\left[{\text{Ca}}^{2+}\right]}_{e}$ provides a lowpass-filtered copy of *x*, while voltage feedback from the soma supplies *z*. The product *xz* determines whether depression or potentiation occurs, depending on its sign.

The brief (1 ms) positive pulses in the glutamate (and GABA) pathways represent discrete release events and are best interpreted as impulse-like event markers. Consequently, the relevant effect of the first-order lowpass filters is not to introduce a transport delay, but to transform each event into a smooth, exponentially shaped trace with time constant τ = *RC*. This filtered trace begins at the event time and provides a slower “eligibility-like” signal that can overlap with other slow variables (e.g., calcium-related signals and feedback) to drive stable weight changes. In contrast, the fast glutamate component is retained to preserve rapid synaptic signaling.

The complete equivalent circuit for the neuron can be formed by combining the circuits illustrated in Figures 4, 6. However, before proceeding to show that the complete circuit implements an adaptive filter, the next subsection offers a brief review of such filters.

### Internal structure and operation of an adaptive filter

**Figure 7:**
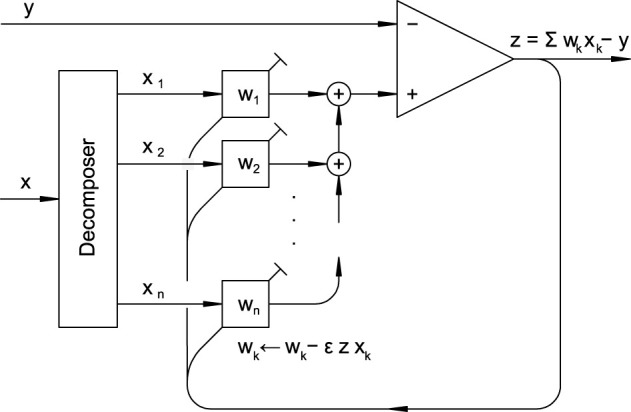
Structure of an adaptive filter (Haykin and Widrow, 2003). A box with input *x*_*k*_ and tunable weight *w*_*k*_ computes the product *x*_*k*_*w*_*k*_ and corresponds to the excitatory synapse in Figure 6. The feedback *z* is crucial for adjusting the weights. The image is a graph representation of Algorithm 1.

This subsection concisely reviews the fundamental adaptive filter (Figure 7), which can be thought of as a procedure or algorithm. Its principal function is to find weights *w*_1_, *w*_2_, …, *w*_*n*_ such that the weighted sum Σ*w*_*k*_*x*_*k*_ of candidate or component signals *x*_1_, *x*_2_, …, *x*_*n*_ approximates a reference signal *y*. The component signals may originate from different sources or be derived from a single input *x* using a delay line or a filter bank as a signal decomposer.

The adaptive filter can be interpreted in different ways, depending on one's perspective.

On the one hand, a biologist might see it as a system that maintains a balance between excitatory and inhibitory inputs (Klyachko and Stevens, 2006; Páscoa dos Santos and Verschure, 2022; Dorrn et al., 2010; Sun et al., 2010). Notably, this differs from homeostasis because the inhibitory-excitatory balance adjusts synaptic weights so that the current weighted excitatory inputs match the inhibitory inputs as well as possible. It is important to note that, although there are multiple inhibitory inputs, the inhibitory weights are fixed according to Gray's rules. Therefore, we can represent the weighted sum of all inhibitory inputs as a single scalar variable *y*. In the adaptive-filter interpretation, learning adjusts the excitatory weight vector *w* so as to reduce the prediction error *z* by matching a weighted sum of excitatory inputs to the inhibitory/reference input. Importantly, this does not imply that the neuron becomes silent for all inputs. The cancellation can only be as good as the representational capacity of the available excitatory inputs: the neuron can reject only those components of the inhibitory signal that lie in the signal space spanned by {*x*_*k*_}. Components of the inhibitory input that cannot be represented as $\underset{k}{\sum }w_{k}x_{k}\left(t\right)$ necessarily remain in the error *z*. When coupled to a spike-generation stage, these residual components naturally yield selective spiking responses to particular input patterns—namely, patterns that are not predicted (or not representable) by the learned excitatory combination. Thus, “balance” in this framework should be understood as cancellation of predictable components within the excitatory input subspace, rather than universal suppression of the output. In signal-processing terms, the circuit performs *rejection* of the predictable subspace and passes the residual.

On the other hand, a physicist might view the filter as performing a wavelet transform of the signal *y* using wavelets *x*_*k*_ (Mallat and Peyré, 2009), with the weights serving as transform coefficients.

Depending on how the filter is connected, it can perform a variety of essential signal processing tasks such as model creation, inverse model creation, prediction, and interference cancellation (Haykin, 2002). Particularly relevant for biological systems is a configuration suggested to address the sensorimotor association problem, or the process by which the brain learns which neuron is connected to which muscle (Nilsson, 2016).

The Least Mean Squares (LMS) algorithm (Algorithm 1) (Haykin and Widrow, 2003), also known as the Widrow-Hoff LMS rule, is a method for updating the weights of an adaptive filter. It operates in discrete time steps *t* = *t*_1_, *t*_2_, …, where at each step it calculates the error feedback *z*, which is the difference between the weighted sum of the input signals *x*_*k*_ and the reference signal *y*. Then, it updates all the weights *w*_*k*_ by subtracting the associated feedback corrections, which are calculated as Δ*w*_*k*_ = ε*zx*_*k*_, where ε is a learning rate. This learning rate is a positive constant, and its selection involves a balance between the convergence speed and stability against noise.

Algorithm 1 The basic LMS algorithm. 
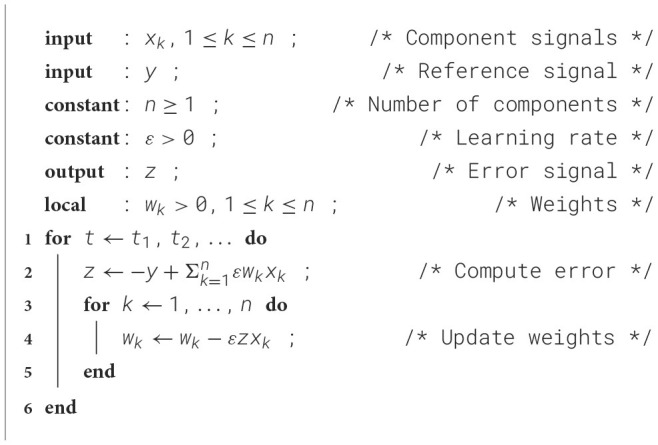

The convergence of the adaptive filter can be understood intuitively as follows: Suppose that some weight *w*_*j*_ is slightly too large and that the corresponding input *x*_*j*_ is positive. Then the error *z* will also tend to be positive and will be fed back to cause a reduction of the weight *w*_*j*_ by ε*zx*_*j*_. A similar argument can be used when instead *w*_*j*_ is too small or *x*_*j*_ is negative. Proving the convergence of the weights formally can be difficult in general, but the LMS rule has proven to be robust in practical applications (Haykin, 2002; Widrow and Stearns, 1985).

### Understanding the neuron as an adaptive filter

Here, it is established that the neuron's equivalent circuit operates as an adaptive filter, suggesting that the neuron also embodies this functionality.

#### The neuron's equivalent circuit as an adaptive filter

**Figure 8:**
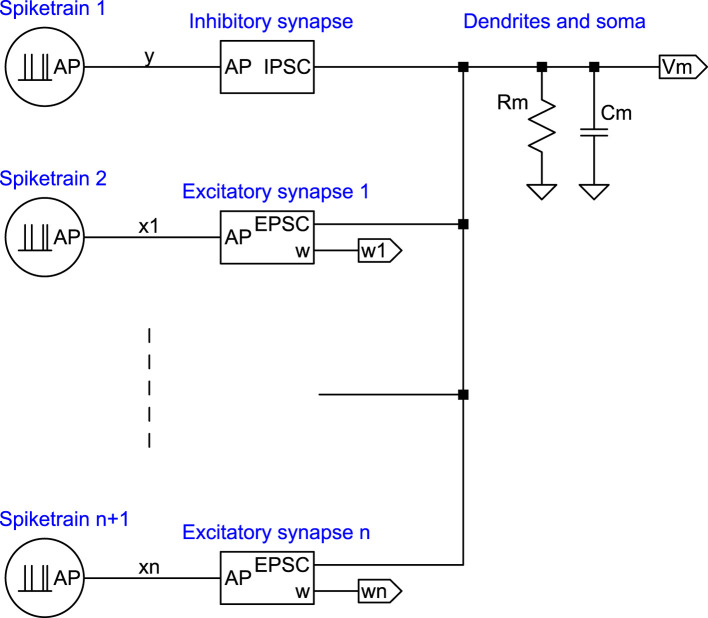
The neuron equivalent circuit. The IPSC and EPSC blocks are defined in Figures 4, 6, respectively. Labels *y, x*1, …, *xn* denote unfiltered action potential (spiketrain) inputs, which are lowpass filtered in the synapse blocks. In the simulations, these inputs should be interpreted as aggregate synaptic drive (i.e., effective lumped spike-train inputs representing many convergent synapses of the same type; see Section Experiment design). The subsequent conversion of *V*_*m*_ back to an output spiketrain and application of an activation function are described elsewhere (Nilsson and Jörntell, 2021).

Interpreting the neuron as an adaptive filter is greatly simplified by modeling the neuron as an equivalent electric circuit. The combination of the synapse circuits in Figures 4, 6 into a circuit equivalent for the neuron is shown in Figure 8. This circuit converts the spiketrain input to membrane potential. The subsequent output conversion of the membrane potential to an output spiketrain and the application of an activation function φ(*z*) are omitted here because a mechanistic model for them has been presented elsewhere (Nilsson and Jörntell, 2021) and does not directly influence the input conversion.

Electrotonic signal propagation is assumed rapid over distances, such as the dendritic tree, while adhering to the RC constraints of the transmission path. Signal processing theory dictates that the feedback loop delay must be significantly shorter than the period of the maximum frequency transmitted through the circuit to maintain stability. Although other mechanisms could be involved in distal signal transmission, their presence is neither evident nor necessary for a mechanistic explanation.

The side-by-side comparison of the adaptive filter, as shown in Figure 7 and the neuron model presented in Figure 8 offers detailed agreement, indicating that both the circuit and, by extension, the neuron implements a modified LMS rule (Algorithm 2). The match between the circuit and the adaptive filter is corroborated below by illustrating how the circuit realizes the summation operations, error feedback, and weight updates. Furthermore, an explanation is provided for the scenario where component inputs are redundant or linearly dependent, a common condition for biological neurons.

Algorithm 2 The modified LMS algorithm, full neuron version with activation function included. The inputs are assumed to already be lowpass filtered. 
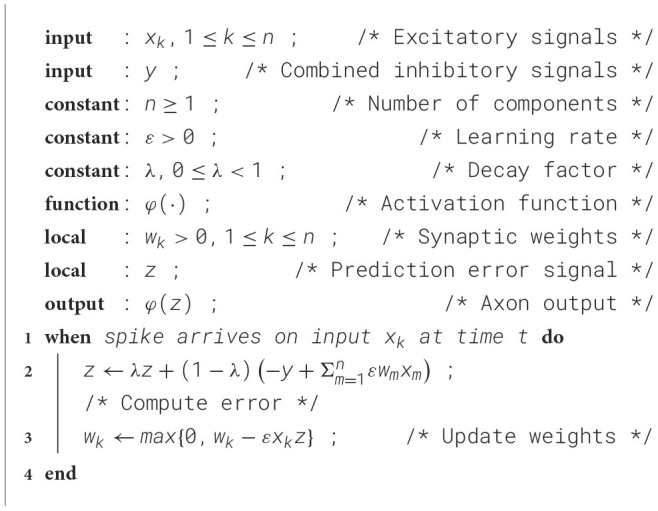

**Summation operations** When comparing the functional blocks in Figure 7 with those in Figure 8, it is evident that the summation operations in the adaptive filter align with the addition of currents in the neuron's equivalent circuit, as Kirchhoff's law dictates. This law states that the sum of currents entering a junction must equal the sum of currents leaving it, mirroring the summation process in the adaptive filter.

**Error feedback** A rapid error feedback signal, labeled by *z* in Figures 6, 7, is essential for the functioning of the adaptive filter, as is visible in Algorithms 1, 2. This feedback is provided by the membrane potential *V*_*m*_ created by the total of the IPSC and EPSC currents passing through the impedance consisting of the membrane resistance *R*_*m*_ in parallel with the membrane capacitance *C*_*m*_. The feedback signal accesses all synapses within the neuron via their connections to the soma. The lowpass filtering by *R*_*m*_*C*_*m*_ introduces a decay or “forget” factor λ, 0 ≤ λ < 1, on line 2 of Algorithm 2, slightly generalizing upon Algorithm 1, which would have λ = 0.

Rongala et al. (2021) have proposed that the membrane capacitance and resistance function as a lowpass filter, stabilizing external feedback in recurrent neural networks. This function is equally applicable to single neurons with internal feedback. In the diagram in Figure 8, this lowpass filter is represented by *R*_*m*_*C*_*m*_, and its impact is encapsulated in the decay factor λ. Notably, this parameter is essential but was not included in the original formulation of LMS learning.

In the biological neuron, the feedback signal *z* is the membrane voltage deviation, which spreads rapidly throughout the dendrite-soma cable by electrotonic conduction. Synaptic inputs enter locally as currents (EPSCs/IPSCs) injected into the dendritic compartment and are summed by the membrane capacitance, whereas the resulting voltage deviation propagates back along the dendrites and is therefore available as a fast, cell-wide postsynaptic state signal at all synapses. Notably, the model does not require the postsynaptic neuron's generation of an action potential to adjust synaptic weights. The membrane potential provides the feedback. This is important because otherwise, a neuron with zero synaptic weights would have difficulties leaving this state.

**Weight updates** The adaptive filter updates its weights *w*_*k*_ by the product of the inputs *x*_*k*_ and the error feedback *z*. The update uses a clever trick that stands out when viewing the involved circuitry, i.e., the plasticity circuitry of the excitatory synapse in Figure 6. The weight *w* is a charge held by the capacitor *C*_*w*_. The product of the input *x* and error *z* should update this weight. However, whereas the error is readily available in the circuit as the membrane potential *V*_*m*_, the signal *x* on the glutamate pathway is PFM encoded and is unusable for the update in this form. Although lowpass filtered in the dendrite and soma, it is directly summed into the membrane potential and is unavailable separately. *Fortunately, a lowpass-filtered version of* *x* *is available as the calcium concentration* ${\left[{\text{Ca}}^{2+}\right]}_{e}$ *in the synaptic cleft*. Thanks to this additional copy of *x*, the NMDAR transistor in the circuit and the ion channel in the neuron can crucially “compute”—pass a charge proportional to—the weight update by multiplying the calcium concentration representing *x* with the membrane potential *V*_*m*_ representing *z*. Experiment 1, described below, validates the above process.

**Redundant and linearly dependent candidate inputs** Decomposing a signal *x* into components in engineering contexts relies on techniques such as a bandpass-filter bank or a Fast Fourier Transform. These methods ensure orthogonality, or at least linear independence, of the components *x*_*k*_. This independence is a critical requirement to guarantee the uniqueness of the weights. As a contrast, such a systematic decomposition is unfeasible from a biological perspective, resulting in identical reference inputs possibly giving rise to different synaptic weights. In the case of redundant component inputs, weights will converge (settle) toward a linear subspace rather than a specific point. Correlated component inputs can slow the convergence of the original LMS algorithm. This is because weights are updated simultaneously, which may lead to overshooting and oscillations. Here, evolution has provided an elegant solution for neurons because each synapse is updated individually and asynchronously by its own glutamate strobe signal (Figure 6), demonstrated in experiment 2 (cf. the “for” statement in Algorithm 1 with the “when” statement in Algorithm 2).

#### Implications of the neuron operating as an adaptive filter

The neuron behaving as an adaptive filter allows us to address the three key concerns in the introduction: the process of information storage and retrieval, the combination of Hebbian and homeostatic plasticity, and the establishment of a unifying rule for synaptic plasticity. The proposed solutions to these problems are presented in the Results section below. More generally, the adaptive filter provides a valuable conceptual model for understanding neuron populations and facilitates a succinct mathematical representation of these (Nilsson, 2023).

The following subsection conducts a series of experiments that confirm the functioning of the circuit as an adaptive filter.

### Experiment design

Four experiments were carried out to explore and test model properties.

**Figure 9:**
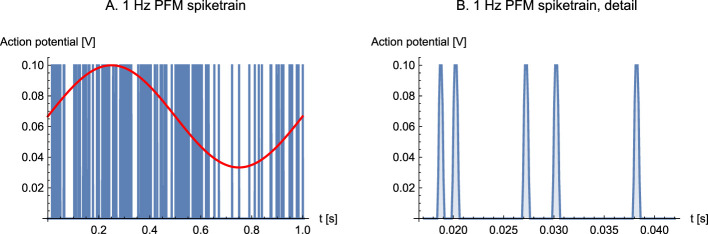
Input signals. **(A)** Initial section of 1 Hz PFM spiketrain used as input. The overlaid sine wave shows the modulation signal. **(B)** Detail. The spike width is 1 ms.

In the first experiment, the stability and convergence of the model were examined. The neuron model comprised one inhibitory synapse and two excitatory synapses (*n* = 2 in Figure 8). The task of the circuit was to determine the weights *w*_1_ and *w*_2_ so that the weighted sum of spiketrains 2 and 3 corresponded to spiketrain 1. The inputs were Pulse Frequency Modulated (PFM) spiketrains, effectively inhomogeneous Poisson processes, modulated by sine waves with a modulation depth of 67% (Figure 9).

The mean spike frequency was 100 Hz, implying an instantaneous minimum and maximum of 33 Hz and 167 Hz, respectively. This baseline pulse rate should not be interpreted as sustained firing of a single presynaptic neuron. Rather, it is a compact surrogate for the aggregate event stream produced by many converging presynaptic sources. A useful intuition, familiar from peri-event raster plots and peri-event (stimulus) time histograms (PSTHs), is that when many neurons are modulated by the same event-locked signal, the population PSTH reveals a smooth modulation even though each single-neuron raster is sparse: pooling many such sparse rasters produces a denser event stream whose rate follows the same envelope.

Formally, when presynaptic spike trains can be approximated as independent inhomogeneous Poisson processes with a shared modulation envelope, their superposition is again an inhomogeneous Poisson process with the same modulation and with baseline rate equal to the sum of individual rates [superposition/merge property (Last and Penrose, 2017)]. Since this theorem belongs to advanced mathematical statistics, we emphasize the practical interpretation: many low-rate inputs can be represented by a single higher-rate pulse train with the same modulation envelope.

This aggregation is most direct for the inhibitory pathway in the reduced circuit, which explicitly represents a combined reference input. For the excitatory pathway the interpretation is less obvious because individual synapses carry adaptive weights; however, Experiment 2 verifies that the excitatory population behaves consistently at the aggregate level (e.g., the learned weights sum to the expected effective weight under modulation), supporting the use of a higher baseline pulse rate as a proxy for convergent physiological drive.

In the simulations, the circuit in Figure 8 should also be understood as a lumped representation at the synapse level: each modeled “synapse” corresponds to the aggregate effect of many biological synapses of the same type converging onto the postsynaptic compartment. This is compatible with the present small-signal (AC) formulation because synaptic inputs enter as currents that sum linearly, and the resulting membrane-voltage deviation likewise superposes in the passive regime.

Consequently, a population of similar synapses driven by independent presynaptic spike trains can be represented by an equivalent circuit of the same topology driven by an aggregate event stream, with parameters interpreted as effective population-level gains. Importantly, this does not imply convergence of multiple neurons onto a single presynaptic membrane; it is a postsynaptic lumping of many distinct terminals.

Sine waves with frequencies that are integer multiples of each other were chosen as stimuli because highly efficient signal processing methods exist to detect and separate such signals embedded in noise. The modulations for the first experiment were 1 Hz and 2 Hz for spiketrains 2 and 3, respectively. The reference input, spiketrain 1, began with a modulation of 1 Hz but switched to 2 Hz after 150 s, ensuring a large number of spike arrivals and NMDA activation episodes. The signal switching after 150 s demonstrates the circuit's responsiveness and detects if convergence to a particular value is merely accidental.

The second experiment aimed to study the model's behavior in the presence of redundant input. In addition, it tested the adequacy of the synapse circuit model for representing not only a single synapse but an aggregate of synapses. Here, “redundancy” refers to the signal-processing identifiability sense: when several excitatory inputs carry overlapping information (i.e., their signals are correlated or span the same subspace), there are generally many different weight vectors that can produce the same net prediction and thus compensate the inhibitory/reference input equally well. The learning problem is under-determined in that case and the model does not have a unique “correct” allocation of weights across those inputs; it converges to one solution within an equivalence class, depending on initialization and any implicit bias of the update dynamics.

In this experiment, a sine wave of 1 Hz modulated the inhibitory input, and a wave of 2 Hz modulated the first excitatory input (*x*_1_). The remaining five excitatory synapses *x*_2_, …*x*_6_ (*n* = 6 in Figure 8) received redundant input. During the first run, these inputs were synchronized, receiving the same spiketrain modulated at 1 Hz. In the second run, spiketrains 3–7 were modulated by 1 Hz but generated independently, mimicking the behavior of biological neurons, making them asynchronous, i.e., spikes arriving independently even though representing the same sine wave.

The component values used in these experiments are provided in Table 1, and they roughly align with physiological values (Hille, 2001). The γ parameters can be measured indirectly by their influence on the speed of adaptation and other time constants. In particular, the γ_NMDAR_ parameter is a lumped parameter that can be tuned to adjust the learning range ε over a wide range. The gamma parameters control the sensitivities of ion channels to gating parameters, such as neurotransmitter concentration. The primary gamma parameter γ_NMDAR_ directly controls calcium influx and thus learning speed. By adjusting this parameter, the neuron can regulate learning, setting it to zero to stop learning or to a high value for rapid learning. This parameter is likely to vary significantly depending on the state and type of neuron. While small calcium currents or gamma values would not cause unphysiological behavior, large values might. However, the experiments demonstrate that the circuit remains stable and functional even with substantial gamma values.

**Table 1:** Model parameters.

**Parameter**	**Value**
*E*_Na_−*E*_quiesc_	130 mV
*E*_Ca_−*E*_quiesc_	190 mV
*E*_Cl_−*E*_quiesc_	-10 mV
γ_GABAAR_	10^−6^ A/V
γ_Ca_V_	10^−8^ A/V
γ_AMPAR_	10^−3^ A/V^2^
γ_NMDAR_	2·10^−5^ A/V^2^
*C*_*h*_, *C*_*h*1_, *C*_*h*2_	10 nF
*R*_*z*_, *R*_*z*1_, *R*_*z*2_, *R*_*h*_	100 MΩ
*C*_*i*_, *C*_*e*_, *C*_*s*_	100 pF
*C*_*w*_	1 nF
*R*_*i*_, *R*_*e*_, *R*_*s*_	1 GΩ
*R*_*m*_	20 MΩ
*C*_*m*_	500 pF

The γ parameters denote ion channel (transistor) gains. Parameters roughly align with physiological values (Hille, 2001).

The reversal potential for chloride ions is close to the quiescent potential at the inhibitory synapse, leading to a small electrochemical driving force for chloride ions, but this poses no issue, as it can be compensated for by a higher γ_GABAAR_ gain. Learning is typically considerably slower in biological neurons, but regardless, the circuit is robust and not overly sensitive to parameter variations.

The third experiment demonstrates recovery from excessively large excitatory weights. In experiment 1, the excitatory synaptic weights start at zero. In experiment 3, the excitatory weights are instead initialized to large values by setting the initial voltages of the *C*_*w*_ capacitors. This test illustrates that the same learning rule that potentiates weights when appropriate also automatically reduces (depresses) them when they are excessive, and that the dynamics still converge. This experiment uses the same inhibitory and excitatory input spiketrains as experiment 1.

The fourth experiment visualizes the time course of the prediction-error feedback *z* during learning. To make the evolution of the prediction error *z* (i.e., the membrane-potential deviation from baseline) easier to interpret, experiment 4 explicitly displays its bidirectional dynamics during adaptation. Because *V*_*m*_ is noisy on the pulse timescale, a lowpass-filtered “probe” attached to *V*_*m*_ is used to reveal a smoothed trajectory of *z* as learning progresses. This experiment uses the same input setup as experiment 3 and compares the time evolution of the errors for both zero and large initial values.

The experiments were conducted using the LTspice electronic-circuit simulator (LTspice XVI, 2022; Engelhardt, 2015). All files needed to reproduce the LTspice simulations are available at the link provided in the Supplementary material.

## Results

### Experiment results

**Figure 10:**
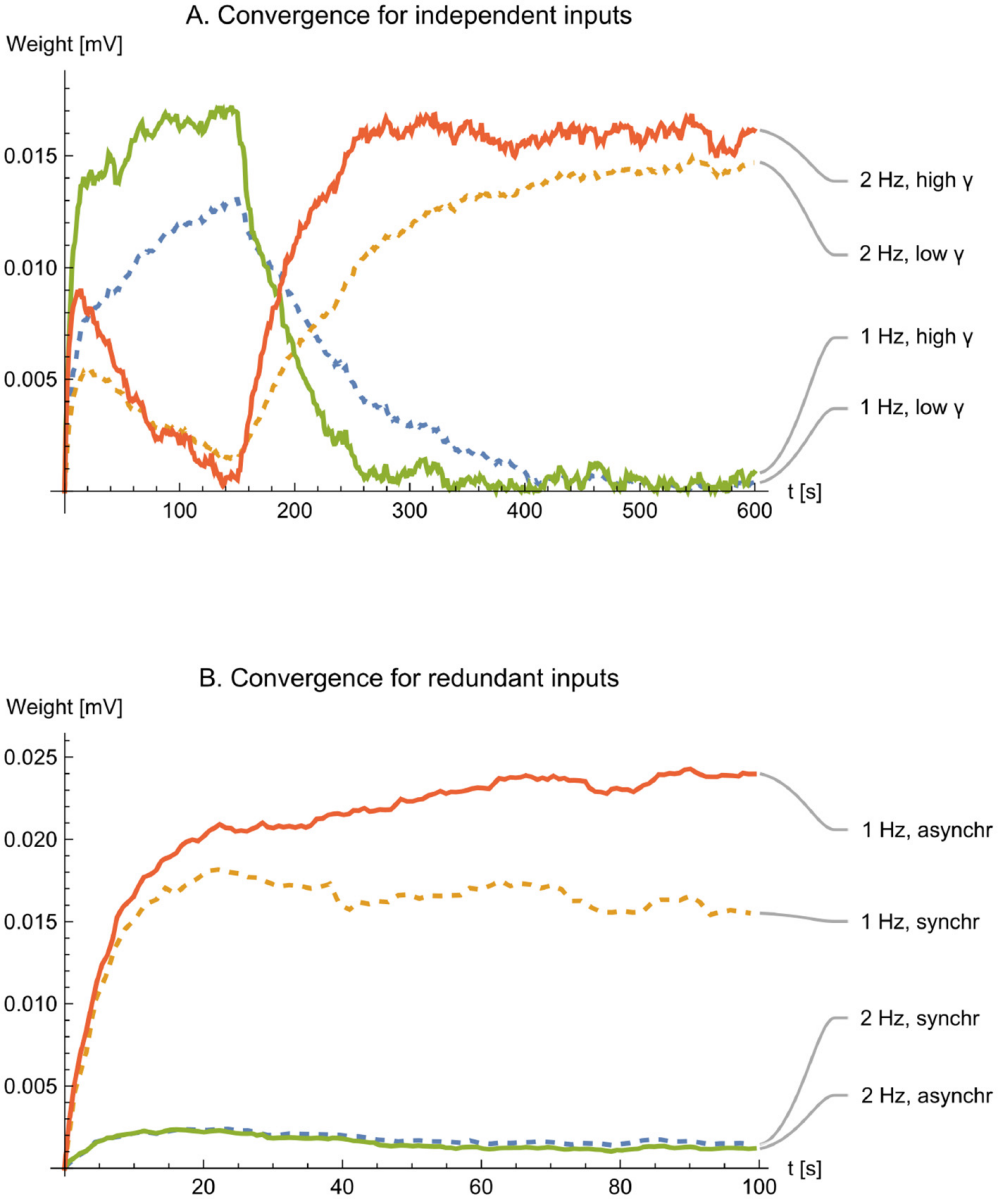
Convergence and stability of weights. **(A)**. Simulations show the convergence of the weights for two different values of NMDAR gain γ (dashed trace for γ = 2·10^−5^ *AV*^−2^ and solid for γ = 5·10^−5^ *AV*^−2^). Modulation of the reference signal changed from 1 Hz to 2 Hz at *t* = 150 s. **(B)**. Convergence for redundant inputs. The upper two traces show the sum *w*_2_+*w*_3_+…+*w*_6_, whereas the lower traces show *w*_1_. Weights cannot be negative. Dashed and solid traces are shown for synchronous and asynchronous spiketrains, respectively.

The first experiment demonstrates the convergence of weights *w*_1_ and *w*_2_. Initially, with the inhibitory input signal *y* modulated by a sine wave of 1 Hz, the ratio *w*_2_/*w*_1_ approaches zero as it should. This is because the input signal *x*_1_ is also modulated by a sine wave of 1 Hz, coinciding with the reference input, while the input signal *x*_2_ is modulated by a sine wave of 2 Hz, which is orthogonal to *y*. However, after 150 s, the modulation of *y* changes to 2 Hz, which instead coincides with the input signal *x*_2_. This time, the inverse ratio *w*_1_/*w*_2_ approaches zero. Figure 10A depicts this convergence for two different values of NMDAR gain γ_NMDAR_. Low and high gain correspond to 2·10^−5^*A*/*V*^2^ and 5·10^−5^*A*/*V*^2^, respectively. The diagram shows that the circuit strives to enhance the weight of the excitatory input that aligns in frequency with the inhibitory input, while concurrently decreasing the weight of the other excitatory input that doesn't match in frequency.

The second experiment shows what happens for multiple redundant excitatory inputs. In computer implementations of adaptive filters, simultaneous switching of redundant inputs can cause instability at high adaptation rates because of overcompensation. In biological neurons, action potentials typically arrive at different synapses asynchronously. Despite that, the experiments show that instability does not occur easily, even for synchronous arrivals.

The parameter γ controls the effective learning rate of the weight-update dynamics: increasing γ increases the magnitude of each update step and therefore accelerates adaptation, whereas decreasing γ slows the rate of convergence. As in standard adaptive-filter learning rules, this introduces a trade-off between convergence speed and stability. In the limit γ → 0, the convergence remains stable but may become too slow to observe on a finite simulation window. Conversely, for sufficiently large γ, the update steps become too large, leading to overshoot and eventual loss of convergence (instability). The two γ values shown in Figure 10 were chosen to illustrate this trade-off, with the larger value selected close to the onset of instability while still exhibiting convergence.

In the first case, all the excitatory inputs are identical, so all strobe pulses are synchronous (dashed traces in Figure 10B). In the second case, the same sine wave of 2 Hz modulates the excitatory inputs, but otherwise, they are independent, so the strobe pulses are asynchronous (solid traces). The experiment shows faster convergence for asynchronous strobes.

**Figure 11:**
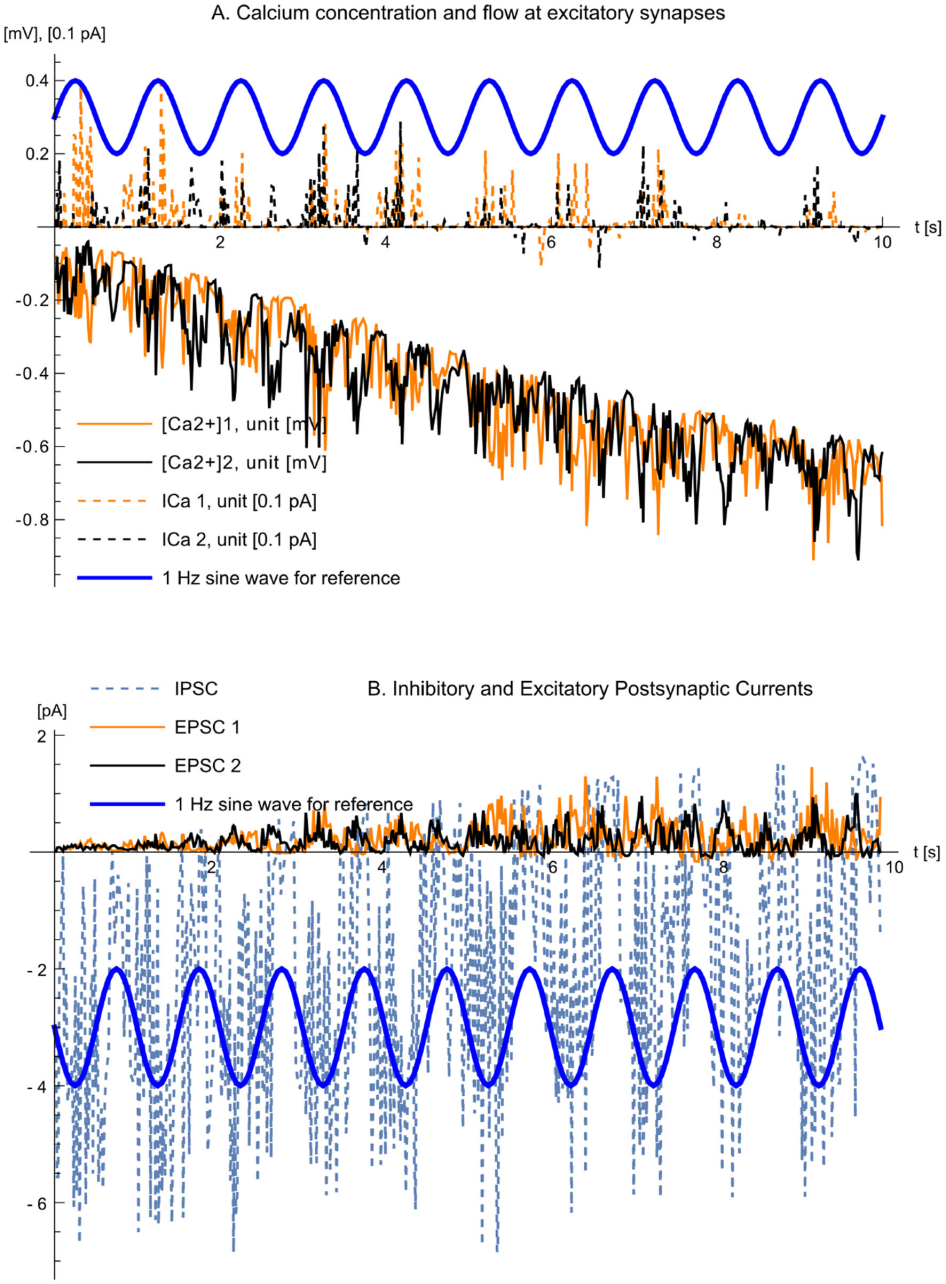
Calcium concentration, flow, IPSC, and EPSC. The figure illustrates the evolution during the first 10 seconds of experiment 1 **(A)**. Representations of ${\left[\text{C}{\text{a}}^{2+}\right]}_{e}$ in the synaptic cleft (lower two traces) and Ca^2+^ flow through the NMDARs. The calcium concentrations are represented as voltages in the model, thus plotted in units of [mV], whereas the calcium currents are plotted in units of [0.1 pA] **(B)**. IPSC and EPSC for the inhibitory and the two excitatory synapses. The blue thick traces are 1-Hz sine waves shown for reference only. The time axes of the panels are aligned.

Figure 11 shows the evolution of synaptic cleft calcium concentration, calcium flow, IPSC, and EPSC during the first ten seconds of experiment 1. The bottom traces in panel A represent the ${\left[\text{C}{\text{a}}^{2+}\right]}_{e}$ at the left terminal of *R*_*s*_ in the synaptic cleft for the two excitatory synapses. Despite some noise, the sine wave modulation of the input signals is evident, and the decrease in synaptic cleft calcium due to presynaptic activity is clearly visible. The top traces show the calcium flow into the NMDARs, where the amplitude of current variations decreases as the cell adjusts synaptic weights to balance inhibitory and excitatory inputs. Panel B illustrates the IPSC, reflecting the 1 Hz modulated input signal, while the EPSCs gradually increase from zero to counterbalance the larger IPSC.

Figure 11A shows the small-signal deviation of cleft calcium from a quiescent operating point, not the absolute concentration. Processes that set and maintain the baseline steady state (diffusion/exchange with surrounding tissue and active clearance) are absorbed into the operating point and are therefore not explicitly modeled. Consequently, after stimulation the plotted calcium variable relaxes back toward zero deviation (quiescence) with the model's characteristic time constants; it should not be interpreted as an unbounded decline of absolute cleft calcium concentration.

It should be noted that understanding the entire neuron's behavior based on individual currents is challenging, as its function involves feedback and relies on the cumulative effects of many small currents over time. It is easier to grasp the cell's behavior through more abstract representations, such as the circuit equivalent in Figure 6, or at the algorithmic level in Algorithms 1, 2. This is a key takeaway of this article.

**Figure 12:**
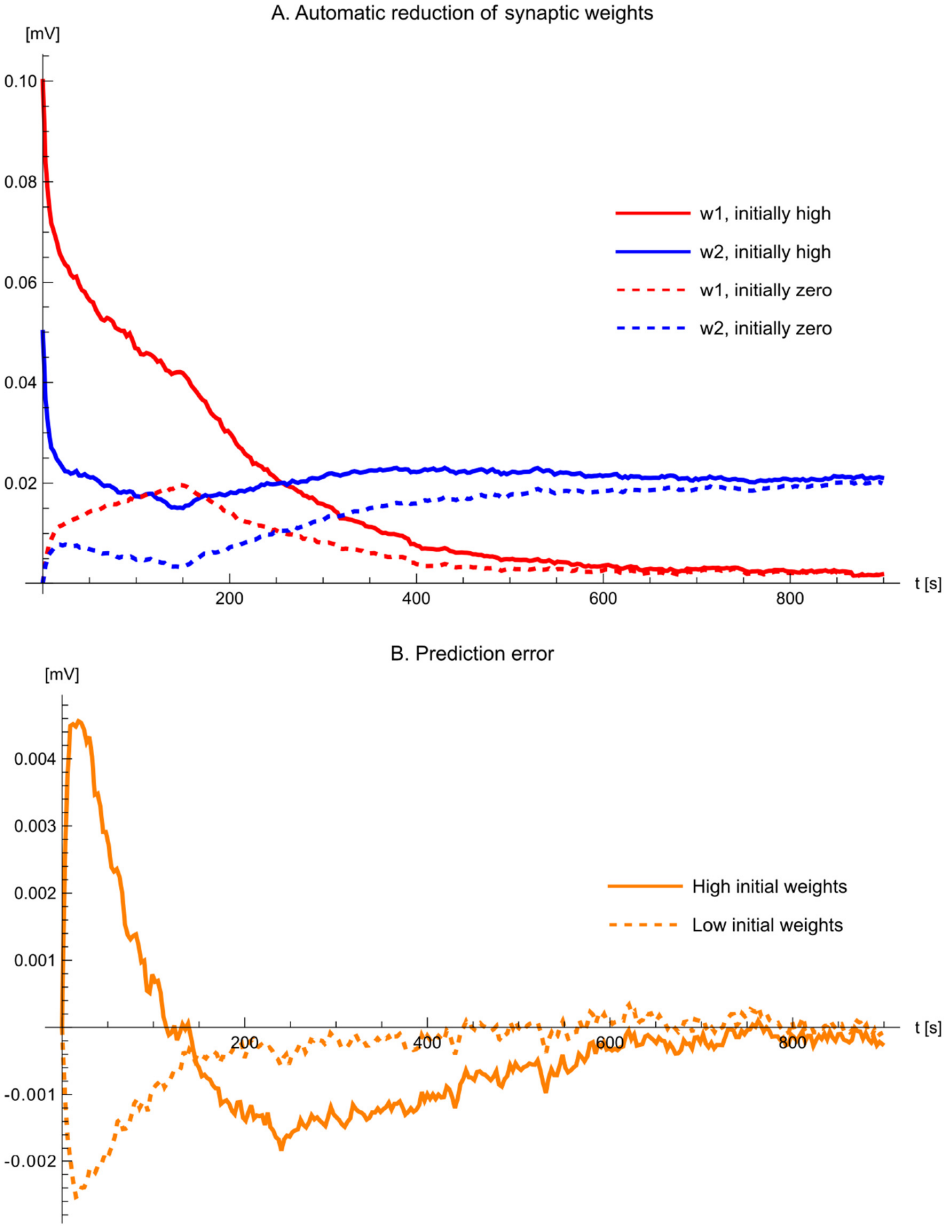
Bidirectional evolution of weights (*w*_1_ and *w*_2_) and prediction error (*z*). The figure shows the first 900 s of experiments 3 and 4. **(A)** The weights converge to the same values whether they are initialized at zero (dashed traces) or at high values (solid traces). **(B)** The prediction-error trajectory *z* is shown for the zero-weight initialization (dashed trace) and the high-weight initialization (solid trace). Note that *z* exhibits bidirectional dynamics and can take both positive and negative values.

The third experiment demonstrates that the excitatory weights converge to the same steady-state values irrespective of initialization (Figure 12A). In particular, weights that start from overly large values are automatically reduced, while weights that start from zero increase when needed, showing that the same update rule supports both potentiation and depression within a single, self-consistent mechanism. The pronounced corner at *t* = 150 s coincides with the programmed change in the inhibitory modulation from 1 Hz to 2 Hz; the subsequent re-convergence illustrates that the learned weights track changes in the reference statistics rather than merely settling to an accidental fixed point.

The fourth experiment visualizes the lowpass-filtered trajectory of the prediction error *z* (Figure 12B), i.e., the membrane-potential deviation from baseline that provides the global feedback signal in the learning rule. Unlike the synaptic weights (which are constrained to remain non-negative), *z* is inherently signed and therefore assumes both negative and positive values, reflecting whether the current weighted excitatory drive over- or under-predicts the inhibitory reference input. Although the instantaneous *z* signal is noisy on the pulse timescale, the lowpass “probe” reveals that its mean trajectory evolves smoothly over learning. This is the relevant quantity for stability: as adaptation proceeds, the smoothed error decreases in magnitude and fluctuates around zero, causing the average update term *zx*_*k*_ to diminish. In this way, the feedback remains informative and sufficiently smooth to guide stable weight convergence even in the presence of stochastic spiking variability.

### Solutions to the three specific problems considered

This paper has suggested that a neuron functions and can be conceptualized as an adaptive filter with internal feedback. Such a neuron model enables straightforward solutions, presented below, to the three problems posed in the introduction.

#### How does the neuron store and retrieve information?

In this adaptive-filter framework, excitatory synaptic weights constitute the model's long-term state variables and therefore encode information about past inputs. The weight vector *w* comprises these long-lived synaptic parameters, implemented here as the number of AMPARs at each synapse.

More precisely, when the reference input is provided as an inhibitory signal *y*(*t*), learning adjusts the weights *w*_*k*_ so that *y*(*t*) is approximated by the weighted sum $\underset{k}{\sum }w_{k}x_{k}\left(t\right)$ of the excitatory component signals *x*_*k*_(*t*), thereby reducing the prediction error *z*. After learning, this stored information can be read out in two complementary ways: (i) when *y*(*t*) and *x*_*k*_(*t*) are again present, the neuron outputs the prediction error *z*, which depends on the learned weights; or (ii) if *y*(*t*) is temporarily held at its baseline, the neuron outputs its current prediction $\underset{k}{\sum }w_{k}x_{k}\left(t\right)$ assembled from the same learned linear combination.

#### Is there a unifying synaptic learning rule?

The synaptic learning rule can be expressed as a variation of the Least Mean Squares (LMS) learning rule, modified to allow asynchronous weight updates, lowpass filtering of the feedback error, and the constraint that weights cannot be negative (cf. line 3 of Algorithm 2):


$$\begin{array}{c} w_{k}\leftarrow \max\left(0,w_{k}-\varepsilon zx_{k}\right), \end{array}$$ (3)

where *k* indicates synapse *k*, $w={\left(w_{1},\ldots ,w_{n}\right)}^{T}$ is a vector denoting the numbers of AMPARs (synaptic weights), *z* represents the lowpass-filtered membrane potential *V*_*m*_ (error feedback), and the vector $x={\left(x_{1},\ldots ,x_{n}\right)}^{T}$ signifies the vectors of local synaptic cleft calcium concentrations ${\left[{\text{Ca}}^{2+}\right]}_{e}$ (excitatory input). The learning rate ε depends on several biological parameters but is perhaps most directly controlled by the gain γ of the NMDAR. This rule is applicable for an arbitrary number of asynchronous inputs and is triggered on a spike arrival at excitatory input *k*.

#### How do homeostatic and Hebbian plasticity balance?

The Hebbian-homeostatic balance emerges from the synaptic learning rule (Equation 3), inherently providing stability and subsuming both Hebbian and homeostatic plasticity. This learning rule attempts to minimize the mean square error between the desired output and the model's prediction. It adjusts the synaptic weights based on the error signal which is the difference between the desired response and the actual output of the adaptive filter.

The stability of the modified LMS algorithm, irrespective of the sign of the input, comes from its inherent structure. The update rule is dependent on the product *zx*_*k*_ of the error *z* and the input *x*_*k*_. The multiplication *z*·*x*_*k*_ is directly implemented by the NMDAR. Even if the input changes sign, the direction of the weight update (whether to increase or decrease the weight) still appropriately aligns with the reduction of the overall error. This is because the error will also adjust its sign based on whether the prediction is above or below the desired outcome. Therefore, the product effectively guides the weight adjustments toward the direction that reduces the error, maintaining the stability of the learning process. Because the parameters *x*_*k*_ and *z* describe the signed deviations from the steady-state averages (homeostatic equilibria), the modified LMS rule offers automatic stabilization.

A potential source of confusion is to interpret the error feedback *z* as a separate homeostatic process that would counteract (“erase”) associative weight changes. In the present framework, this is not the case: there is only one update rule, and the error signal *z* serves as a gate and sign for synapse-specific plasticity through the product *z*·*x*_*k*_. The factor *x*_*k*_ ensures input specificity (only active synapses update), while the factor *z* ensures that updates occur only when the neuron's current prediction mismatches the reference. Consequently, learned weights are not driven back to baseline; instead, learning is self-limiting because as the mismatch decreases, *z* decreases and the update term *zx*_*k*_ vanishes. Stabilization is therefore inherent to the error-correcting structure of the rule rather than imposed by an additional homeostatic plasticity mechanism.

## Discussion

### The neuron as a differential element

The neuron uses membrane potential feedback during adaptation to adjust the excitatory synapse weights. This adjustment strives to balance inhibitory and excitatory input. Importantly, the feedback does not pull weights toward a preset value; it encodes the instantaneous mismatch between the neuron's prediction and the reference. When that mismatch is small, the feedback is small and weight updates cease, which is the mechanism by which stability arises.

Alternatively, this process can be described as the neuron's attempt to predict the inhibitory input by excitatory input—the membrane potential encodes the *prediction error* (Schultz and Dickinson, 2000). Signal processing and control theory often refer to prediction error as the fundamental concept *innovation* (Kailath, 1968). It has frequently been discussed in neuroscience under different names, including *novelty* (Kohonen, 1977), *unexpectedness* (Barlow, 1991), *decorrelation* (Dean et al., 2002), *surprise* (Friston et al., 2006), and *saliency* (van Polanen, 2014).

The critical operation for the plasticity of the neuron is the multiplication of the prediction error feedback *z*, represented by the membrane potential *V*_*m*_, with the excitatory input *x* available from the synaptic cleft external calcium concentration ${\left[{\text{Ca}}^{2+}\right]}_{e}$. Given the existence of this non-linear multiply mechanism, linear mechanisms can adjust a suitable homeostatic equilibrium or zero offset (*x*_0_, *z*_0_) by processes involving voltage-gated calcium channels (*zx*_0_) and metabotropic glutamate receptors (*z*_0_*x*).

A significant difference between a neuron and a classical adaptive filter is that the neuron's weights cannot be negative. This is not a limitation because feeding a candidate signal *x* together with its negation (−*x*) achieves the same effect as a signed weight (Dean et al., 2013). Incorporating such negations could be a function of the numerous local inhibitory neurons in the nervous system. Somewhat unexpectedly, this restriction to non-negative weights proves to be an advantage, as it enhances the expressive capabilities of neuron *populations* (Nilsson, 2023).

### Relation to neuromorphic engineering

Synapses have long been modeled as equations (Roth and van Rossum, 2009; Urbanczik and Senn, 2014), and particularly in the field of neuromorphic engineering, as electronic circuits (Schuman et al., 2017). These models are predominantly empirical, but they are typically too detailed in some respects and lack other crucial aspects to be useful for a mechanistic explanation of plasticity. This should not be construed as a dismissal of empirical models because they are significant in the development of neuroscience. Biologically inspired VLSI circuits are foundational, e.g., in achieving computational performance in neuromorphic engineering, and deserve recognition, even when biologically implausible. The specific concept that neuronal synapses function as lowpass filters, with an input spike typically resulting in a current shaped like an alpha function, has been a standard in neuron modeling. It has been systematically described by Gerstner and Kistler (2002) but is not easy to attribute to any individual because it has evolved through cumulative research in the field.

Most circuit elements used here were introduced in neuron modeling well before the term “neuromorphic engineering” was coined in the late 1980s. The concept of the RC circuit as a foundation for neuron models was first proposed empirically by Lapicque (1907), and later, Cole and Curtis (1939) developed it from a mechanistic perspective based on the bilayer structure of the membrane. Hodgkin and Huxley (1952) suggested the existence of gated ion channels, which effectively functioned as transistors, although they were not depicted as such due to the unfamiliarity with transistors at the time.

Braeken et al. (2009) constructed a FET transistor directly gated by glutamate. Dutta and Roy (2011) introduced a variation of the Hodgkin-Huxley model where ion-sensitive FET transistors represent synapses. The Gerstner and Kistler (2002) description of the NMDA receptor aligns with the approach taken here, but their work is strictly confined to mathematical equations and does not involve introducing electronic components.

### Low-level model properties

Two salient features which distinguish the proposed model are the explicit dynamics of the synaptic cleft and the dual-purpose utilization of glutamate for both direct information transfer and as a strobe signal that facilitates weight adjustment. The necessity for a strobe input arises because if NMDARs were continuously active, weights would be diluted toward zero, resulting in information loss. It is crucial for plasticity that weights change only when there is meaningful input—that is, when activated by glutamate (Huganir and Nicoll, 2013).

The circuit equivalent assumes that NMDARs operate at the same speed as AMPARs. In reality, NMDARs are slower and produce a burst of openings when triggered by glutamate, effectively performing a lowpass filtering. The model does not explicitly incorporate this property because the lowpass-filtered calcium input already accounts for the slowdown.

Several researchers have put forth adaptive filters as models for neuronal circuits in the cerebellum, utilizing *external* feedback (Fujita, 1982; Wolpert et al., 1998; Porrill et al., 2013). Nevertheless, low-latency feedback is pivotal for the performance of an adaptive filter as it sets the maximum signal frequency content. External feedback is slower than internal feedback by several orders of magnitude (for pyramidal neurons, see, e.g., Mihaljević et al., 2021; Antic, 2003).

The idea of a neuron functioning as a self-contained adaptive filter has been hypothesized (Nilsson, 2016; Luczak et al., 2022). However, the model presented here appears to be the first wholly mechanistic model based exclusively on the known properties of ion channels.

While the chloride reversal potential acts as a limiter for large signal deflections and hyperpolarizations, this function is not crucial for mechanistically explaining plasticity when conducting a small-signal analysis. It is worth noting that the difference between the membrane potentials and the chloride reversal potential measured under physiological conditions *in vivo* tends to be greater than what the more common *in vitro* measurements suggest. More broadly speaking, in this model, the inhibitory synapse lacks plasticity and serves the simple role of signal inversion, lowpass filtering, and introducing an IPSC. A simple circuit can adequately model this functionality by a straightforward inverter followed by a lowpass filter, as detailed in Supplementary material Equations 1–8.

The present paper adopts Gray's rules (Gray, 1959) only as a modeling prior, i.e., as a pragmatic asymmetry that guides where the model's explicit learning mechanism is placed. This prior is motivated by the well-known anatomical association between excitatory synapses and dendritic spines, which are strongly linked to synaptic plasticity, and by the frequent proximal localization of inhibitory synapses on shafts or soma. Importantly, this is not a biological claim that inhibitory synapses are intrinsically non-plastic: inhibitory synapses are well known to express plasticity, including neuromodulator-gated forms, through induction pathways that are diverse and context dependent. Because such mechanisms do not provide a single, canonical “NMDA-like” coincidence gate that would uniquely specify a rapid, voltage-dependent learning rule in the present framework, we treat inhibitory efficacies as effectively constant on the modeled time window and restrict explicit weight updates to excitatory synapses. Extending the model to inhibitory plasticity would therefore require introducing and justifying a separate inhibitory induction/update rule; while similar circuit-analytic techniques may still be applicable, that extension is beyond the scope of the present work.

For the studied GABAAR-AMPAR-NMDAR neurons, the model assumes that signals are conveyed by minor deviations from equilibrium. The general approach to model neurons by circuit equivalents can certainly also be applied in more general cases involving large deviations and steep changes in ion channel conductance depending on the operating conditions of the neuron, but in such cases, it will most likely be harder to find as simple an abstraction as the update rule (Equation 3).

### High-level model properties

Most neuronal plasticity experiments seem to apply uniform stimuli to both inhibitory and excitatory inputs. However, this study suggests that these inputs should be treated differently, as synaptic weight changes are heavily influenced by the relationship between them. Differentiating the stimuli for inhibitory and excitatory inputs is likely one of the most significant experimental proposals arising from this study.

A central prediction of the model is that the learning rate ε, or metaplasticity parameter, is directly related to the gain of the external-calcium-to-AMPAR cascade reflected by the lumped parameter γ_NMDAR_. Two types of interrelated experiments on real neurons could test this prediction. The first would test whether such a parameter is conceivable, e.g., by modifying the most convenient and accessible factor influencing the learning rate. The second would more exhaustively attempt to identify the factors affecting the gain and their interrelations.

One likely candidate for influencing the metaplasticity is the baseline concentration of external calcium (Dunwiddie and Lynch, 1979; Turner et al., 1982; Inglebert et al., 2020; Inglebert and Debanne, 2021; Gaviño et al., 2015). Research, including a study using knockout mice (Nishiyama et al., 2002), suggests that astrocytes regulate this concentration, significantly impacting LTP and LTD. Conveniently for experimentation, other studies have demonstrated that astrocyte activity can be modulated by noradrenaline (Wahis and Holt, 2021), providing a potential experimental pathway for further investigation.

Conducting a sensitivity analysis to measure the factors influencing the learning rate is challenging because the above lumped-parameter gain of the NMDA receptor summarizes this sensitivity. Many factors influence this parameter, providing neurons with multiple adjustment methods. This adaptability is advantageous for the neuron, as it can select the most beneficial adjustment method. However, this complexity and the compensatory nature of these factors result in a broad operating range for each factor, making it hard to pinpoint parameter values.

When interpreting the circuits in Figures 4, 6 from an electrical engineering perspective, it appears that evolution has crafted a robust and minimalist solution. From a pure signal processing standpoint, the stability of neuronal functions strongly suggests the existence of feedback. The loop delay in this feedback must be short, pointing toward electrotonic propagation. Within the present model class and time scale, the membrane potential is the only readily available fast postsynaptic state signal: output spikes are too sparse to provide comparably swift, continuously valued feedback.

The neuron seems to use the biochemical equivalent of alternating current (AC) signals for communication, while the direct current (DC) level is regulated by homeostasis to maintain a suitable metabolic balance. It is hard to imagine a more efficient configuration of components capable of performing such a complex signal-processing task. Evolution has produced an elegant solution, utilizing current summation for feed-forward processes and voltage for feedback. The dual role of the glutamate pulse, acting both as a pulse-frequency modulated input and a strobe, is particularly striking.

Widrow and Hoff (1960) initially introduced the abstract, high-level neuron model ADALINE (for ADAptive LInear NEuron), drawing inspiration from the McCulloch-Pitts neuron model (McCulloch and Pitts, 1990). This work predates the experimental discovery of ion channels by several years. Regrettably, Widrow and Hoff eventually abandoned ADALINE as a neuron model. Nevertheless, it became the foundation of the adaptive filter, which experienced dramatic advancements within the signal processing domain.

The LMS learning rule is known under various names in different contexts. In the field of artificial neural networks, it is often referred to as the “delta rule,” whereas in statistical learning theory, as the “covariance rule” (Sejnowski, 1977). These names all refer to the same concept: an iterative method for adjusting the weights of a learning model to minimize the mean square error ||*z*|| between the model's prediction, which is the weighted sum of *x*_*k*_, and the actual data *y*. The proposed model is a mechanistic explanation of a modified LMS or covariance rule with asynchronous updates, restricted to non-negative weights and including a decay factor. Other major self-stabilizing learning rules are the Bienenstock-Cooper-Munro (BCM) rule (Bienenstock et al., 1982) and the Oja rule (Oja, 1982). However, these rules are theoretical constructs and, to the best of the author's knowledge, lack mechanistic explanations.

The proposed model, when compared to biological neurons, exhibits several characteristics typical of biological neurons but not commonly found in other neuron models, at least not mechanistic ones:

1. It possesses the ability to record time-variable functions.
2. The model can learn without risking instability. This and the previous feature align with two of the three fundamental properties we initially aimed to achieve, as outlined in the introduction.
3. The capacity to “bootstrap” from a state where all synapse weights are zero is difficult for neurons relying on output spikes for plasticity.

The presented model does not include the process by which the neuron converts the membrane potential into the output spiketrain, including the activation function, because this process has been comprehensively addressed in a recent publication, which mechanistically explains this output process (Nilsson and Jörntell, 2021). The current paper completes the picture of the neuron by providing a mechanistic explanation of the input process—the conversion back to internal potential from spike trains, including the plasticity.

### Related forms of plasticity

There is a vast body of literature exploring the mechanisms behind LTP and LTD, with many models focusing on the fine details of biophysical processes underlying synaptic plasticity. These models often aim to capture the intricate biochemical pathways that mediate calcium entry and AMPAR recruitment, but despite the level of detail in these studies, none, to the best of the author's knowledge, provide a unified, mechanistic explanation of the neuron's plasticity as a whole. This is largely due to the fact that these models operate at an overly specific level, focusing on the minutiae of chemical pathways where consensus is still lacking.

What is generally accepted, on the other hand, is the fundamental relationship that increased calcium levels lead to an increase in the number of AMPARs. This is the abstraction level at which the current model operates. While simplified, it effectively captures the core dynamic between calcium influx and synaptic weight modulation. This straightforward relationship, as demonstrated, is sufficient for explaining the broader behavior of the neuron.

In contrast to more detailed models that focus on replicating the specific biochemical pathways involved in LTP and LTD, this model offers a higher-level perspective that provides a mechanistic explanation of the neuron's learning process. The goal is parsimony, not minimal biology: Calcium is included as the canonical biochemical trigger for plasticity, but we avoid introducing additional unconstrained plastic parameters (e.g., an inhibitory learning rule) that are not required to explain the phenomenon addressed here.

Several computational studies of synaptic plasticity acknowledge the importance of calcium current through NMDARs (Shouval et al., 2002; Rackham et al., 2010; Graupner and Brunel, 2012). These models tend to focus heavily on spike-timing-dependent plasticity (STDP) and overlook the role of external calcium concentration, which complicates the acquisition of presynaptic activity (Graham et al., 2014).

It has been shown experimentally that STDP is not required for plasticity (Isaac et al., 1995; Liao et al., 1995), though it remains compatible with the model. A postsynaptic spike generates backward-propagating fluctuations in the membrane potential. While often called a backward-propagating action potential (BPAP), this spike is heavily lowpass filtered, appearing distally as a depolarization followed by hyperpolarization. If this “backwash” coincides with presynaptic activity, it can lead to an increase or decrease in synaptic weight, depending on the relative timing, as it contributes to the voltage error feedback, but a full analysis of this effect is beyond the scope of this paper and would require separate research.

Several other neuronal features have been discussed and speculatively related to plasticity, including electrical effects of the spine neck (Harnett et al., 2012), location (Saudargienė and Graham, 2015), intraspine action potentials (Plotkin et al., 2013), and shunting of synaptic currents by simultaneously active synapses on a single spine (Keller, 2002). As for spine neck effects that passive filters can characterize, they benefit the neuron by increasing the diversity of synapse filter characteristics. However, the proposed model is generally independent of exotic features. Standard features of ion channels are entirely satisfactory for explaining all aspects of the model. Neither are exotic features deleterious for the model, as it is robust against noise in its capacity as an adaptive filter.

## Conclusions

Neuroscience research in many fields depends on detailed mechanistic knowledge of how neurons decode, process, store, and encode information. Examples of such fields are neural implants, interoception, and artificial intelligence, but progress in these fields has struggled with empirical and oversimplified neuron models.

This manuscript provides a self-contained model of the synaptic input-to-membrane-potential transformation (including plasticity); combined with the membrane-potential-to-spike-output model in (Nilsson and Jörntell 2021), it yields a complete neuron model in the signal-processing sense. The model is not intended to include all known biological plasticity mechanisms, but rather the minimal set required to explain the plasticity phenomenon addressed here. The model explains at the ion channel level how neurons convert input spiketrains to internal potential, including the adjustments of their synaptic weights. Crucial components of the model are the inclusion of synaptic cleft dynamics, the arrangement of internal feedback, and the multiple functions of the glutamate neurotransmitter. It is shown that information storage can be identified with the weight adjustments of an adaptive filter. The neuron strives to balance the inhibitory and excitatory inputs. After adaptation, it can be regarded as an inhibitory input predictor, delivering the prediction error as output.

The mechanistic abstraction of the neuron as an adaptive filter constitutes an essential link to the realm of conceptual spaces (Gärdenfors, 2000) interposed between the cognitive and biological levels. It reduces the need for spiking-level simulations and simplifies the understanding of large assemblies and networks of neurons, elaborated in-depth in Nilsson (2023).

## Data Availability

The datasets presented in this study can be found in online repositories. The names of the repository/repositories and accession number(s) can be found in the article/Supplementary material.

## References

[B1] Antic S. D. (2003). Action potentials in basal and oblique dendrites of rat neocortical pyramidal neurons. J. Physiol. 550, 35–50. doi: 10.1113/jphysiol.2002.033746 12730348 PMC2343022

[B2] Barlow H. B. (1991). Representations of Vision: Trends and Tacit Assumptions in Vision Research, chapter Vision tells you more than “What is Where.” Cambridge: Cambridge University Press, 319–329.

[B3] Bienenstock E. Cooper L. Munro P. (1982). Theory for the development of neuron selectivity: orientation specificity and binocular interaction in visual cortex. J. Neurosci. 2, 32–48. doi: 10.1523/JNEUROSCI.02-01-00032.1982 7054394 PMC6564292

[B4] Bliss T. V. P. Collingridge G. L. (1993). A synaptic model of memory: long-term potentiation in the hippocampus. Nature 361, 31–39. doi: 10.1038/361031a0 8421494

[B5] Bliss T. V. P. Lømo T. (1973). Longlasting potentiation of synaptic transmission in the dentate area of the anaesthetized rabbit following stimulation of the perforant path. J. Physiol. 232, 331–356. doi: 10.1113/jphysiol.1973.sp010273 4727084 PMC1350458

[B6] Borst J. G. G. Sakmann B. (1999). Depletion of calcium in the synaptic cleft of a calyxtype synapse in the rat brainstem. J. Physiol. 521, 123–133. doi: 10.1111/j.1469-7793.1999.00123.x 10562339 PMC2269650

[B7] Braeken D. Rand D. R. Andrei A. Huys R. Spira M. E. Yitzchaik S. . (2009). Glutamate sensing with enzyme-modified floating-gate field effect transistors. Biosens. Bioelectr. 24, 2384–2389. doi: 10.1016/j.bios.2008.12.012 19155170

[B8] Cohen J. E. Fields R. D. (2004). Extracellular calcium depletion in synaptic transmission. Neuroscientist 10, 12–17. doi: 10.1177/1073858403259440 14987443 PMC6660135

[B9] Cole K. S. Curtis H. J. (1939). Electric impedance of the squid giant axon during activity. J. General Physiol. 22, 649–670. doi: 10.1085/jgp.22.5.649 19873125 PMC2142006

[B10] Craver C. F. (2006). When mechanistic models explain. Synthese 153, 355–376. doi: 10.1007/s11229-006-9097-x

[B11] Craver C. F. (2007). Explaining the Brain. Oxford: Oxford University Press. doi: 10.1093/acprof:oso/9780199299317.001.0001

[B12] Dean P. Anderson S. Porrill J. Jörntell H. (2013). An adaptive filter model of cerebellar zone C3 as a basis for safe limb control? J. Physiol. 591, 5459–5474. doi: 10.1113/jphysiol.2013.261545 23836690 PMC3853489

[B13] Dean P. Porrill J. Stone J. V. (2002). Decorrelation control by the cerebellum achieves oculomotor plant compensation in simulated vestibulo-ocular reflex. Proc. R. Soc. London Series B 269, 1895–1904. doi: 10.1098/rspb.2002.2103 12350251 PMC1691115

[B14] Dorrn A. L. Yuan K. Barker A. J. Schreiner C. E. Froemke R. C. (2010). Developmental sensory experience balances cortical excitation and inhibition. Nature 465, 932–936. doi: 10.1038/nature09119 20559387 PMC2888507

[B15] Dunwiddie T. V. Lynch G. (1979). The relationship between extracellular calcium concentrations and the induction of hippocampal long-term potentiation. Brain Res. 169, 103–110. doi: 10.1016/0006-8993(79)90377-9 222396

[B16] Dutta J. Roy S. (2011). Modeling neuron for simulation of transmitter gated ion channels of postsynaptic membrane at synaptic cleft. Am. J. Biomed. Sci. 3, 176–182. doi: 10.5099/aj110300176

[B17] Engelhardt M. (2015). SPICE differentiation. Technol. J. Analog Innov. 24, 10–16. Available online at: www.analog.com/media/en/technical-documentation/lt-journal-article/LTJournal-V24N4-2015-01.pdf

[B18] Friston K. Kilner J. Harrison L. (2006). A free energy principle for the brain. J. Physiol.-Paris. 100, 70–87. doi: 10.1016/j.jphysparis.2006.10.001 17097864

[B19] Fujita M. (1982). Adaptive filter model of the cerebellum. Biol. Cybern. 45, 195–206. doi: 10.1007/BF00336192 7171642

[B20] Gärdenfors P. (2000). Conceptual Spaces: the Geometry of Thought. Cambridge, MA: MIT Press. doi: 10.7551/mitpress/2076.001.0001

[B21] Gaviño M. A. Ford K. J. Archila S. (2015). Homeostatic synaptic depression is achieved through a regulated decrease in presynaptic calcium channel abundance. Elife 4:e05473. doi: 10.7554/eLife.05473 25884248 PMC4443758

[B22] Gerstner W. Kistler W. M. (2002). Spiking Neuron Models: Single Neurons, Populations, Plasticity. Cambridge Univ. Press, New York. doi: 10.1017/CBO9780511815706

[B23] Graham B. P. Saudargiene A. Cobb S. (2014). Spine head calcium as a measure of summed postsynaptic activity for driving synaptic plasticity. Neural Comput. 26, 2194–2222. doi: 10.1162/NECO_a_00640 25058697

[B24] Graupner M. Brunel N. (2012). Calcium-based plasticity model explains sensitivity of synaptic changes to spike pattern, rate, and dendritic location. Proc. Nat. Acad. Sci. 109, 3991–3996. doi: 10.1073/pnas.1109359109 22357758 PMC3309784

[B25] Gray E. G. (1959). Axo-somatic and axo-dendritic synapses of the cerebral cortex: An electron microscope study. J. Anat. 93, 420–433. 13829103 PMC1244535

[B26] Harnett M. T. Makara J. K. Spruston N. Kath W. L. Magee J. C. (2012). Synaptic amplification by dendritic spines enhances input cooperativity. Nature 491, 599–602. doi: 10.1038/nature11554 23103868 PMC3504647

[B27] Harris K. M. Weinberg R. J. (2012). Ultrastructure of synapses in the mammalian brain. Cold Spring Harb. Perspect. Biol. 4:a005587. doi: 10.1101/cshperspect.a005587 22357909 PMC3331701

[B28] Haykin S. Widrow B. (2003). Least-Mean-Square Adaptive Filters. Hoboken, NJ: John Wiley &Sons, Inc. doi: 10.1002/0471461288

[B29] Haykin S. S. (2002). Adaptive Filter Theory. Upper Saddle River, NJ: Prentice Hall.

[B30] Hille B. (2001). Ion Channels of Excitable Membranes. Sunderland, MA: Sinauer Associates.

[B31] Hodgkin A. L. Huxley A. F. (1952). Currents carried by sodium and potassium ions through the membrane of the giant axon of Loligo. J. Physiol. 116, 449–472. doi: 10.1113/jphysiol.1952.sp004717 14946713 PMC1392213

[B32] Horowitz P. Hill W. (1989). The Art of Electronics. Cambridge, UK: Cambridge Univeristy Press.

[B33] Huganir R. L. Nicoll R. A. (2013). AMPARs and synaptic plasticity: the last 25 years. Neuron 80, 704–717. doi: 10.1016/j.neuron.2013.10.025 24183021 PMC4195488

[B34] Inglebert Y. Aljadeff J. Brunel N. Debanne D. (2020). Synaptic plasticity rules with physiological calcium levels. Proc. Nat. Acad. Sci. 117, 33639–33648. doi: 10.1073/pnas.2013663117 33328274 PMC7777146

[B35] Inglebert Y. Debanne D. (2021). Calcium and spike timing-dependent plasticity. Front. Cell. Neurosci. 15:727336. doi: 10.3389/fncel.2021.727336 34616278 PMC8488271

[B36] Isaac J. T. R. Nicoll R. A. Malenka R. C. (1995). Evidence for silent synapses: implications for the expression of LTP. Neuron 15, 427–434. doi: 10.1016/0896-6273(95)90046-2 7646894

[B37] Kailath T. (1968). An innovations approach to least-squares estimation–Part I: linear filtering in additive white noise. IEEE Trans. Automat. Contr. 13, 646–655. doi: 10.1109/TAC.1968.1099025

[B38] Keck T. Toyoizumi T. Chen L. Doiron B. Feldman D. E. Fox K. . (2017). Integrating Hebbian and homeostatic plasticity: the current state of the field and future research directions. Philos. Trans. R. Soc. B 372:20160158. doi: 10.1098/rstb.2016.0158 28093552 PMC5247590

[B39] Keller A. (2002). Use-dependent inhibition of dendritic spines. Trends Neurosci. 25, 541–543. doi: 10.1016/S0166-2236(02)02260-9 12392919 PMC2813855

[B40] Klyachko V. A. Stevens C. F. (2006). Excitatory and feed-forward inhibitory hippocampal synapses work synergistically as an adaptive filter of natural spike trains. PLoS Biol. 4:e207. doi: 10.1371/journal.pbio.0040207 16774451 PMC1479695

[B41] Kohonen T. (1977). “Associative memory,” in Communication and Cybernetics (Berlin Heidelberg: Springer). doi: 10.1007/978-3-642-96384-1

[B42] Lapicque L. (1907). Recherches quantitatives sur l'excitation électrique des nerfs traitée comme une polarization. J. Physiol. Pathol. Gén. 9, 620–635.

[B43] Last G. Penrose M. (2017). Lectures on the Poisson Process. Cambridge: Cambridge University Press. doi: 10.1017/9781316104477

[B44] Liao D. Hessler N. A. Malinow R. (1995). Activation of postsynaptically silent synapses during pairing-induced LTP in CA1 region of hippocampal slice. Nature 375, 400–404. doi: 10.1038/375400a0 7760933

[B45] Lisman J. (2017). Glutamatergic synapses are structurally and biochemically complex because of multiple plasticity processes: long-term potentiation, long-term depression, short-term potentiation and scaling. Philos. Trans. R. Soc. B 372:20160260. doi: 10.1098/rstb.2016.0260 28093558 PMC5247596

[B46] LTspice XVI I. (2022). Version 17.0.34.0 (64-bit). Available online at: https://www.analog.com/en/resources/design-tools-and-calculators/ltspice-simulator.html (Accessed April, 2024).

[B47] Luczak A. McNaughton B. L. Kubo Y. (2022). Neurons learn by predicting future activity. Nat. Mach. Intell. 4, 62–72. doi: 10.1038/s42256-021-00430-y 35814496 PMC9262088

[B48] Mallat S. G. Peyré G. (2009). A Wavelet Tour of Signal Processing: the Sparse Way. Burlington, MD: Elsevier/Academic Press.

[B49] Marder E. Goaillard J.-M. (2006). Variability, compensation and homeostasis in neuron and network function. Nat. Rev. Neurosci. 7, 563–574. doi: 10.1038/nrn1949 16791145

[B50] Mayer M. L. Westbrook G. L. Guthrie P. B. (1984). Voltage-dependent block by Mg2+ of NMDA responses in spinal cord neurones. Nature 309, 261–263. doi: 10.1038/309261a0 6325946

[B51] McCulloch W. S. Pitts W. (1990). A logical calculus of the ideas immanent in nervous activity. Bull. Mathem. Biol. 52, 99–115. doi: 10.1016/S0092-8240(05)80006-0 2185863

[B52] Mihaljević B. Larrañaga P. Bielza C. (2021). Comparing the electrophysiology and morphology of human and mouse layer 2/3 pyramidal neurons with bayesian networks. Front. Neuroinform. 15:580873. doi: 10.3389/fninf.2021.580873 33679362 PMC7930221

[B53] Nelson S. B. Turrigiano G. G. (2008). Strength through diversity. Neuron 60, 477–482. doi: 10.1016/j.neuron.2008.10.020 18995822 PMC4919814

[B54] Nilsson M. (2016). “Neuronal “op-amps” implement adaptive control in biology and robotics,” in Human and Robot Hands: Sensorimotor Synergies to Bridge the Gap Between Neuroscience and Robotics, eds. M. Bianchi, A. Moscatelli (Cham: Springer International Publishing), 69–86. doi: 10.1007/978-3-319-26706-7_6

[B55] Nilsson M. N. P. (2023). Information processing by neuron populations in the central nervous system: mathematical structure of data and operations. arXiv preprint arXiv:2309.02332.

[B56] Nilsson M. N. P. Jörntell H. (2021). Channel current fluctuations conclusively explain neuronal encoding of internal potential into spike trains. Phys. Rev. E 103:022407. doi: 10.1103/PhysRevE.103.022407 33736029

[B57] Nishiyama H. Knöpfel T. Endo S. Itohara S. (2002). Glial protein S100B modulates long-term neuronal synaptic plasticity. Proc. Nat. Acad. Sci. 99, 4037–4042. doi: 10.1073/pnas.052020999 11891290 PMC122644

[B58] Nowak L. Bregestovski P. Ascher P. Herbet A. Prochiantz A. (1984). Magnesium gates glutamate-activated channels in mouse central neurones. Nature 307, 462–465. doi: 10.1038/307462a0 6320006

[B59] O'Brien R. J. Kamboj S. Ehlers M. D. Rosen K. R. Fischbach G. D. Huganir R. L. (1998). Activity-dependent modulation of synaptic AMPA receptor accumulation. Neuron 21, 1067–1078. doi: 10.1016/S0896-6273(00)80624-8 9856462

[B60] Oja E. (1982). Simplified neuron model as a principal component analyzer. J. Math. Biol. 15, 267–273. doi: 10.1007/BF00275687 7153672

[B61] OpenAI (2024). ChatGPT-4. Available online at: https://openai.com/chatgpt (Accessed January 14, 2026).

[B62] Páscoa dos Santos F. Verschure P. F. M. J. (2022). Excitatory-inhibitory homeostasis and diaschisis: tying the local and global scales in the post-stroke cortex. Front. Syst. Neurosci. 15:806544. doi: 10.3389/fnsys.2021.806544 35082606 PMC8785563

[B63] Plotkin J. L. Shen W. Rafalovich I. Sebel L. E. Day M. Chan C. S. . (2013). Regulation of dendritic calcium release in striatal spiny projection neurons. J. Neurophysiol. 110, 2325–2336. doi: 10.1152/jn.00422.2013 23966676 PMC3841873

[B64] Porrill J. Dean P. Anderson S. R. (2013). Adaptive filters and internal models: Multilevel description of cerebellar function. Neural Netw. 47, 134–149. doi: 10.1016/j.neunet.2012.12.005 23391782

[B65] Purves D. Augustine G. J. Fitzpatrick D. Hall W. C. LaMantia A.-S. White L. E. (2012). Neuroscience. Sunderland, MA: Sinauer Associates.

[B66] Rackham O. J. L. Tsaneva-Atanasova K. Ganesh A. Mellor J. R. (2010). A Ca2+-based computational model for NDMA receptor-dependent synaptic plasticity at individual post-synaptic spines in the hippocampus. Front. Synaptic Neurosci. 2:31. doi: 10.3389/fnsyn.2010.00031 21423517 PMC3059685

[B67] Rongala U. B. Enander J. M. D. Kohler M. Loeb G. E. Jörntell H. (2021). A non-spiking neuron model with dynamic leak to avoid instability in recurrent networks. Front. Comput. Neurosci. 15:656401. doi: 10.3389/fncom.2021.656401 34093156 PMC8173185

[B68] Roth A. van Rossum M. C. W. (2009). “Modeling synapses,” in Computational Modeling Methods for Neuroscientists, 139–160. doi: 10.7551/mitpress/9780262013277.003.0007

[B69] Sallard E. Letourneur D. Legendre P. (2021). Electrophysiology of ionotropic GABA receptors. Cell. Molec. Life Sci. 78, 5341–5370. doi: 10.1007/s00018-021-03846-2 34061215 PMC8257536

[B70] Saudargienė A. Graham B. P. (2015). Inhibitory control of site-specific synaptic plasticity in a model CA1 pyramidal neuron. BioSystems. 130, 37–50. doi: 10.1016/j.biosystems.2015.03.001 25769669

[B71] Schultz W. Dickinson A. (2000). Neuronal coding of prediction errors. Annu. Rev. Neurosci. 23, 473–500. doi: 10.1146/annurev.neuro.23.1.473 10845072

[B72] Schuman C. D. Potok T. E. Patton R. M. Birdwell J. D. Dean M. E. Rose G. S. . (2017). A survey of neuromorphic computing and neural networks in hardware. arXiv preprint arXiv:1705.06963.

[B73] Sejnowski T. J. (1977). Storing covariance with nonlinearly interacting neurons. J. Math. Biol. 4, 303–321. doi: 10.1007/BF00275079 925522

[B74] Shouval H. Z. Bear M. F. Cooper L. N. (2002). A unified model of NMDA receptor-dependent bidirectional synaptic plasticity. Proc. Nat. Acad. Sci. 99, 10831–10836. doi: 10.1073/pnas.152343099 12136127 PMC125058

[B75] Sjöström P. J. (2021). Grand challenge at the frontiers of synaptic neuroscience. Front. Synaptic Neurosci. 13:748937. doi: 10.3389/fnsyn.2021.748937 34759809 PMC8575031

[B76] Sun Y. J. Wu G. K. Liu B. Li P. Zhou M. Xiao Z. . (2010). Fine-tuning of pre-balanced excitation and inhibition during auditory cortical development. Nature 465, 927–931. doi: 10.1038/nature09079 20559386 PMC2909826

[B77] Traynelis S. F. Wollmuth L. P. McBain C. J. Menniti F. S. Vance K. M. Ogden K. K. . (2010). Glutamate receptor ion channels: structure, regulation, and function. Pharmacol. Rev. 62, 405–496. doi: 10.1124/pr.109.002451 20716669 PMC2964903

[B78] Turner R. W. Baimbridge K. G. Miller J. J. (1982). Calcium-induced long-term potentiation in the hippocampus. Neuroscience 7, 1411–1416. doi: 10.1016/0306-4522(82)90254-8 6126840

[B79] Turrigiano G. G. (2017). The dialectic of Hebb and homeostasis. Philos. Trans. R. Soc. B 372:20160258. doi: 10.1098/rstb.2016.0258 28093556 PMC5247594

[B80] Turrigiano G. G. Leslie K. R. Desai N. S. Rutherford L. C. Nelson S. B. (1998). Activity-dependent scaling of quantal amplitude in neocortical neurons. Nature 391, 892–896. doi: 10.1038/36103 9495341

[B81] Turrigiano G. G. Nelson S. B. (2004). Homeostatic plasticity in the developing nervous system. Nat. Rev. Neurosci. 5, 97–107. doi: 10.1038/nrn1327 14735113

[B82] Urbanczik R. Senn W. (2014). Learning by the dendritic prediction of somatic spiking. Neuron 81, 521–528. doi: 10.1016/j.neuron.2013.11.030 24507189

[B83] van Polanen V. (2014). Findings in Haptic (Re)search. PhD thesis, VU University Amsterdam, ISBN: 978-94-6259-188-2.

[B84] Wahis J. Holt M. G. (2021). Astrocytes, noradrenaline, α1-adrenoreceptors, and neuromodulation: evidence and unanswered questions. Front. Cell. Neurosci. 15:645691. doi: 10.3389/fncel.2021.645691 33716677 PMC7947346

[B85] Widrow B. Hoff M. E. (1960). “Adaptive switching circuits,” in IRE WESCON Convention Record (Los Angeles, CA: Institute of Radio Engineers, Institute of Radio Engineers), 96–104.

[B86] Widrow B. Stearns S. D. (1985). Adaptive Signal Processing. Prentice-Hall signal processing series. Englewood Cliffs, NJ: Prentice-Hall.

[B87] Wolpert D. M. Miall R. C. Kawato M. (1998). Internal models in the cerebellum. Trends Cogn. Sci. 2, 338–347. doi: 10.1016/S1364-6613(98)01221-2 21227230

