# Supplementary material for: Mechanistic explanation of neuroplasticity using equivalent circuits
Source: Front Comput Neurosci. 2026 Feb 13;20:1716559. doi: 10.3389/fncom.2026.1716559 (PMC12946124; doi:10.3389/fncom.2026.1716559)
Supplement: Supplementary file 1 [file Presentation_1.pdf]

Mechanistic Explanation of Neuroplasticity Using Equivalent Circuits – Supplementary Material –

Martin N. P. Nilsson
RISE Research Institutes of Sweden,
P.O.B. 1263, SE-164 29 Kista, Sweden
martin.nilsson@ri.se
ORCID: 0000-0002-7504-0328

Data availability

SPICE netlists (`.asc`), symbol files (`.asy`), plot specifications (`.plt`), and generated data files (`.log` and `.raw`) designed and used during the current study are available in the Zenodo permanent repository at doi:10.5281/zenodo.18368358).

Synapse circuit equations

The equations decribing the inhibitory synapse in Figure 4 are shown below for the purpose of illustrating the complexity of an equation-based approach. Positive directions for currents and voltages are down and to the right.

$$I_{GABAAR} = \gamma_{GABAAR} V_{AP} V_{GABAAR}, \tag{1}$$
$$V_{Rz} = R_z I_{Rz}, \tag{2}$$
$$V_{Rz} = V_{Cl-} + V_{GABAAR}, \tag{3}$$
$$I_{Ci} = C_i \frac{V_{Rz}}{dt}, \tag{4}$$
$$I_{IPSC} = -I_{Rz} - I_{GABAAR} - I_{Ci}, \tag{5}$$
$$V_{Ri} = R_i I_{IPSC}, \tag{6}$$
$$I_{Ch} = C_h \frac{U_{Ch}}{dt}, \tag{7}$$
$$V_{IPSC} = V_{Rz} - V_{Ri} - V_{Ch}. \tag{8}$$

Equation (1) is the same as (2) in the section describing the inhibitory synapse.

The equations corresponding to the excitatory synapse in Figure 6 are

$$I_{AMPAR} = \gamma_{AMPAR} V_{AP} V_{Cw} V_{AMPAR}, \tag{9}$$

$$I_{Ca.V} = -\gamma_{Ca.V} V_{AP} V_{Ca.V}, \tag{10}$$

$$V_{Rz2} = R_{z2} I_{Rz2}, \tag{11}$$

$$V_{Rz2} = -V_{Ca2+} - V_{Ca.V}, \tag{12}$$

$$I_{Cs} = C_s \frac{V_{Rz2}}{dt}, \tag{13}$$

$$I_{Rs} = I_{Ca.V} - I_{Rz2} - I_{Cs}, \tag{14}$$

$$V_{Rs} = R_s I_{Rs}, \tag{15}$$

$$I_{Ch2} = C_{h2} \frac{V_{Ch2}}{dt}, \tag{16}$$

$$V_{Rh} = V_{Rz2} - V_{Rs} - V_{Ch2}, \tag{17}$$

$$V_{Rh} = R_h I_h, \tag{18}$$

$$I_{NMDAR} = I_s - I_{Rh}, \tag{19}$$

$$I_{NMDAR} = \gamma_{NMDAR} V_{AP} V_{NMDAR} V_{EPSC}, \tag{20}$$

$$I_{Cw} = C_w \frac{V_{Cw}}{dt}, \tag{21}$$

$$I_D = I_{NMDAR} - I_{Cw}, \tag{22}$$

$$I_D = I_{D0}(1 - e^{-\alpha V_{Cw}}), \tag{23}$$

$$V_{Rz1} = R_{z1} I_{Rz1}, \tag{24}$$

$$V_{Rz1} = -V_{AMPAR} + V_{Na+}, \tag{25}$$

$$I_{Ce} = C_e \frac{V_{Rz1}}{dt}, \tag{26}$$

$$I_{AMPAR} = I_{Rz1} + I_{Ce} + I_{Re}, \tag{27}$$

$$V_{EPSC} = V_{Rz1} + V_{Re} + V_{Ch1}, \tag{28}$$

$$V_{Re} = R_e I_{Re}, \tag{29}$$

$$I_{Ch1} = C_{h1} \frac{V_{Ch1}}{dt}, \tag{30}$$

$$V_{EPSC} = V_{Rc1} - V_{Re} - V_{ch1}. \tag{31}$$

Complexity is introduced by the four non-linear equations (9), (10), (20), and (23), the five differential equations (13), (16), (21), (26), and (30), and the the feedback coupling by V_{EPSC} in (20).

The solution of the above equations, i.e., the model simulation, is carried out using the SPICE circuit simulator LTspice [2] via a netlist representation of the equivalent circuit. LTspice employs an Ordinary Differential Equation (ODE)

solver with a modified trapezoidal integrator and a variable time step. It incorporates special numerical techniques for handling nonlinear equations, sparse matrices, and implicit integration [1].

The circuit simulator itself is agnostic with regard to neurons and neuroscience, which offers an advantage over specialized neuron simulators as there are no built-in hidden assumptions. The implementation is fully transparent, as all biological information is explicitly provided by the netlist.

Below, some details are provided how a system of non-linear ODEs is solved using the implicit trapezoidal method combined with Newton iterations.

The first step of the simulator is to express the network in the standard form

$$\frac{dy}{dt} = F(y,t), \tag{32}$$

where y is a vector of the unknown currents and voltages. This is a straightforward process accomplished by taking the time derivative of formulas which don't already contain a time derivative. The implicit trapezoidal method then introduces difference approximations of the derivatives and updates the solution from y_n at time t_n to y_{n+1} at time $t_{n+1} = t_n + \Delta t$ using the formula

$$y_{n+1} = y_n + \frac{\Delta t}{2}\left(F(y_n,t_n) + F(y_{n+1},t_{n+1})\right). \tag{33}$$

This equation is implicit because y_{n+1} appears on both sides of the equation, making it a non-linear algebraic equation when $F(y,t)$ is non-linear. Here, Newton iterations are used to solve the implicit equation for y_{n+1}. The equation to solve is

$$G(y_{n+1}) = y_{n+1} - y_n - \frac{\Delta t}{2}\left(F(y_n,t_n) + F(y_{n+1},t_{n+1})\right) = 0. \tag{34}$$

Newton iteration starts with an initial guess $y_{n+1}^{(0)}$ which is the previous time step's solution, $y_{n+1}^{(0)} = y_n$. Iterative updates are then performed to refine the guess $y_{n+1}^{(k)}$ using the Newton iteration formula

$$y_{n+1}^{(k+1)} = y_{n+1}^{(k)} - \left[\left.\frac{\partial G}{\partial y_{n+1}}\right|_{y_{n+1}^{(k)}}\right]^{-1} G(y_{n+1}^{(k)}) \tag{35}$$

Here, $\frac{\partial G}{\partial y_{n+1}}$ is the Jacobian of $G(y_{n+1})$ with respect to y_{n+1}. The current guess $y_{n+1}^{(k)}$ is the substituted into the equation for $G(y_{n+1})$:

$$G(y_{n+1}^{(k)}) = y_{n+1}^{(k)} - y_n - \frac{\Delta t}{2}\left(F(y_n,t_n) + F(y_{n+1}^{(k)},t_{n+1})\right) \tag{36}$$

The Jacobian of $G(y_{n+1})$ with respect to y_{n+1} is given by

$$\frac{\partial G}{\partial y_{n+1}} = I - \frac{\Delta t}{2} \frac{\partial F}{\partial y_{n+1}}\bigg|_{y_{n+1}^{(k)}}, \tag{37}$$

where I is the identity matrix and $\frac{\partial F}{\partial y}$ is the Jacobian matrix of $F(y,t)$ with respect to y. After this, the Newton iteration formula computes the next guess,

$$y_{n+1}^{(k+1)} = y_{n+1}^{(k)} - \left(I - \frac{\Delta t}{2} \frac{\partial F}{\partial y_{n+1}}\bigg|_{y_{n+1}^{(k)}} \right)^{-1} \left(y_{n+1}^{(k)} - y_n - \frac{\Delta t}{2} \left(F(y_n, t_n) + F(y_{n+1}^{(k)}, t_{n+1}) \right) \right). \tag{38}$$

The iteration continues until the change in y_{n+1} between iterations is below a specified tolerance,

$$\|y_{n+1}^{(k+1)} - y_{n+1}^{(k)}\| < \varepsilon, \tag{39}$$

where ε is a small positive number.

References

[1] M. Engelhardt. SPICE Differentiation. *Linear Technology Journal of Analog Innovation*, 24(4):10–16, Jan. 2015. URL: `https://www.analog.com/media/en/technical-documentation/lt-journal-article/LTJournal-V24N4-2015-01.pdf`.

[2] LTspice XVII. Version 17.0.34.0 (64-bit), 2022. (URL visited April, 2024). URL: `https://www.analog.com/en/resources/design-tools-and-calculators/ltspice-simulator.html`.
